# Structural Transformation of the Tandem Ubiquitin-Interacting Motifs in Ataxin-3 and Their Cooperative Interactions with Ubiquitin Chains

**DOI:** 10.1371/journal.pone.0013202

**Published:** 2010-10-07

**Authors:** Ai-Xin Song, Chen-Jie Zhou, Yu Peng, Xue-Chao Gao, Zi-Ren Zhou, Qing-Shan Fu, Jing Hong, Dong-Hai Lin, Hong-Yu Hu

**Affiliations:** 1 State Key Laboratory of Molecular Biology, Institute of Biochemistry and Cell Biology, Shanghai Institutes for Biological Sciences, Chinese Academy of Sciences, Shanghai, China; 2 Shanghai Institute of Materia Medica, Shanghai Institutes for Biological Sciences, Chinese Academy of Sciences, Shanghai, China; Indiana University, United States of America

## Abstract

The ubiquitin-interacting motif (UIM) is a short peptide with dual function of binding ubiquitin (Ub) and promoting ubiquitination. We elucidated the structures and dynamics of the tandem UIMs of ataxin-3 (AT3-UIM12) both in free and Ub-bound forms. The solution structure of free AT3-UIM12 consists of two α-helices and a flexible linker, whereas that of the Ub-bound form is much more compact with hydrophobic contacts between the two helices. NMR dynamics indicates that the flexible linker becomes rigid when AT3-UIM12 binds with Ub. Isothermal titration calorimetry and NMR titration demonstrate that AT3-UIM12 binds diUb with two distinct affinities, and the linker plays a critical role in association of the two helices in diUb binding. These results provide an implication that the tandem UIM12 interacts with Ub or diUb in a cooperative manner through an allosteric effect and dynamics change of the linker region, which might be related to its recognitions with various Ub chains and ubiquitinated substrates.

## Introduction

Ubiquitin (Ub)-interacting motif (UIM) is a highly conserved short peptide sequence found in a number of different proteins and can bind to mono- or poly-ubiquitin (monoUb or polyUb) [1], [2]. It was originally identified from the S5a subunit of the 19S proteasome [3], where the UIM interacts with the ubiquitinated proteins or polyUb chains, and facilitates degradation of the selected proteins by proteasome. UIM motifs are also prevalent in the proteins that function in the pathways of endocytosis and vacuolar protein sorting, like Epsins [4], Eps15 [5], [6] and Hrs (yeast Vps27p) [7], [8], and in the DNA repair pathway, like RAP80 [9]. Ataxin-3 (AT3) is a polyglutamine (polyQ)-containing protein that is related to one kind of the polyQ diseases, spinocerebellar ataxia 3 or Machado-Joseph disease (SCA3/MJD) [10]. It contains two or three conserved UIM motifs around the polyQ tract in sequence that are crucial for binding Ub and promoting ubiquitination of substrates [11], [12].

The conserved residues of UIMs, especially the hydrophobic residues, play important roles in Ub recognition. Similar to other known Ub-binding domains, such as Ub-associated domain (UBA), UIM mainly interacts with the Leu8-Ile44-Val70 hydrophobic patch on Ub molecule [13], [14]. The three-dimensional structures of UIM motifs in several proteins have also been solved [15], [16], [17], [18]. Typically, each UIM motif adopts a single α-helix structure in solution, whereas UIM2 from Vps27p forms a four-helix bundle as an oligomer when crystallized [16] and the two UIMs of RAP80 merge into a long helix when bound with K63-linked diUb [19].

Although UIM is a single-helix motif, it displays various features and diverse functions when it binds to Ub. For instance, the double-sided UIM in Hrs protein can bind two Ub molecules simultaneously on both sides [20]. The sequence of the double-sided UIM, characterized by the overlay of two UIMs (a special form of tandem UIMs), has been proposed as a strategy for increasing its binding efficiency. Another example is MIU (motif interacting with Ub) or IUIM (inverted UIM), which is identified through studies of the Rabex-5 GTPase exchange factor [21], the mammalian ortholog of yeast Vps9p. The helix of Rabex-5 binds across the Ile44 surface of Ub in the same position as does a canonical UIM helix, but with the opposite polarity. This interaction represents another interesting variation in the UIM recognition motif. Notably, many proteins contain multiple copies of the UIM motif connected by linkers with different lengths. The repeat of UIM sequence suggests the presence of possible cooperation in the interactions with Ub chains or ubiquitinated substrates, as in the case of an endocytic protein Cue that binds with Ub [22]. However, the previous studies on S5a and Vps27p proteins did not reveal any cooperative effect, even though both of them contain tandem UIMs [16], [18]. Instead, their individual UIMs bind Ub independently and non-cooperatively.

**Figure 1:**
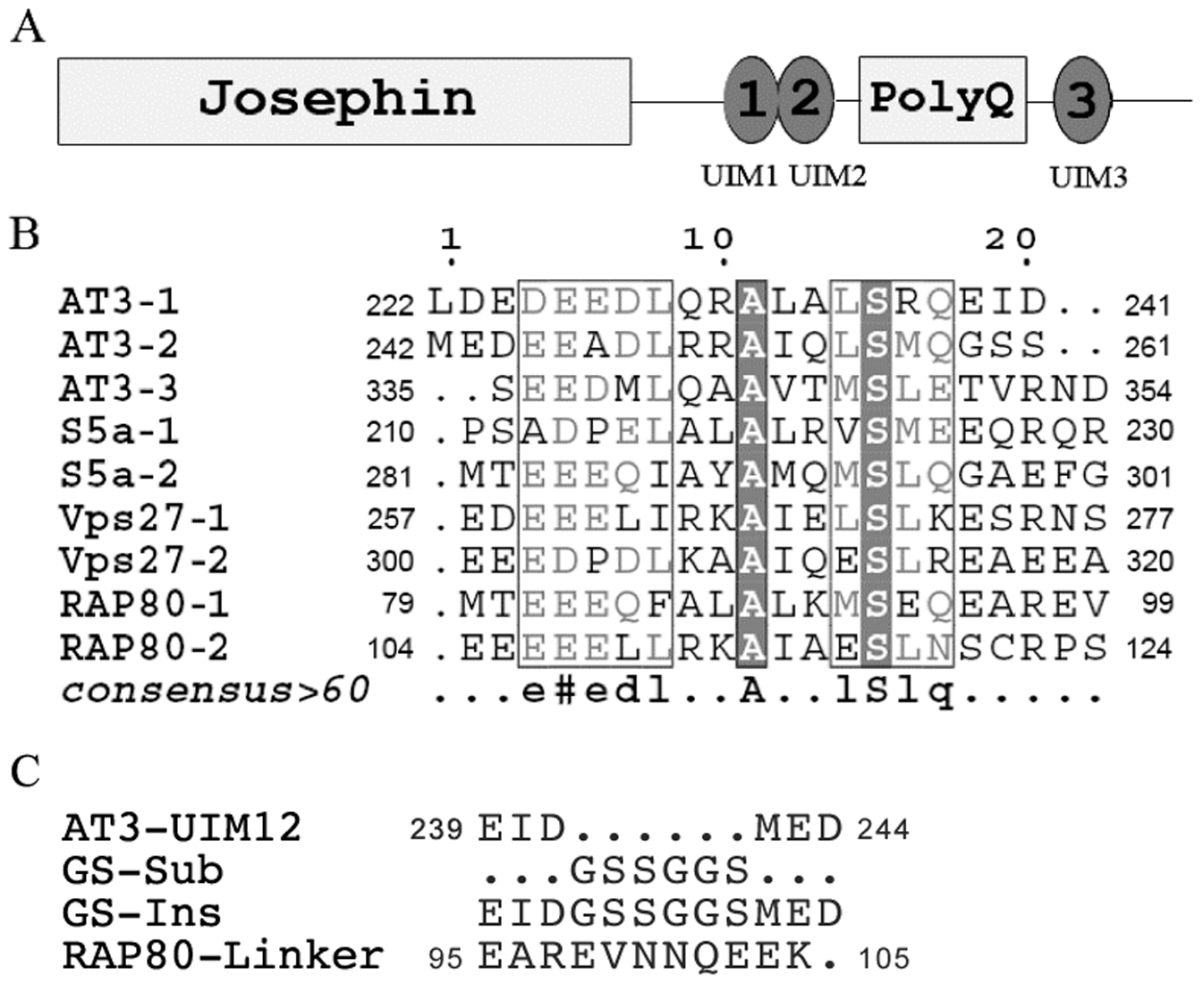
Domain Architecture of AT3 Protein and Sequence Alignments of the UIM Motifs. A, Domain architecture of AT3 protein. B, Multiple sequence alignments of the UIM motifs from AT3, S5a, Vps27 and RAP80. Note that the length of the linker region between the two tandem UIM motifs varies in these four proteins. The linker in AT3 is the shortest (6 residues), and then in RAP80 (∼11 residues), whereas those in S5a and Vps27p are longer with 50 and 23 residues, respectively. The sequences were aligned using ClustalW and represented using ESPript. C, Sequences of the linker regions for wild-type AT3-UIM12 and its mutants. GS-Sub, substitution with GSSGGS sequence; GS-Ins, Insertion of GSSGGS; RAP80-Linker, replacement with the linker sequence of RAP80.

As a 42-kDa multi-domain protein, AT3 consists of an N-terminal compact Josephin domain, two UIM motifs, a polyQ stretch, and a short variable tail (Fig. 1A) [23]. There is another UIM (UIM3) at the C-terminal region, which was only identified in a splice variant [24]. The Josephin domain itself can bind Ub and has deubiquitinating activity, which functions in regulating P97/VCP-associated retrotranslocation [25]. The binding of Ub to AT3 is also mediated by its UIMs, and single-point mutation of the conserved serine residues in the UIM motifs can eliminate its binding to ubiquitinated proteins [11]. Hence, the UIM regions have been proposed to recruit polyubiquitinated substrates to position the polyUb chains adjacent to the catalytic site, and/or to allow the enzyme to act in trimming polyUb chains in a distal to proximal direction [26]. It is reasonable to speculate that multiple copies of UIM would be conducive to this function.

The UIM sequences (∼20 residues) of AT3 are similar to those found in many other proteins, such as S5a, Vps27p, AT3, and RAP80 (Fig. 1B) [15]. These proteins each contain two tandem UIM motifs; however, the linker regions between these UIM motifs are largely different. The UIMs of S5a and Vps27p are interrupted by a long linker of 20∼50 residues, whereas the two UIM motifs of AT3 are juxtapositional to each other in sequence with the shortest linker. This short linker may provide a possibility that the two UIM motifs bind Ub cooperatively.

The Ub binding property of AT3 has linked its normal biological function to protein surveillance pathway [12]. Accumulation of evidence suggests that the Ub-proteasome pathway also participates in the pathogenesis of the diseases [27], [28], [29]. To clarify the specific recognition between tandem UIMs of AT3 (AT3-UIM12) and Ub chains or ubiquitinated substrates that may be of functional significance, we determined the solution structures of AT3-UIM12 in free and bound forms, analyzed its binding properties with Ub or Ub chains by NMR, isothermal titration calorimetry (ITC) and other biochemical techniques. The importance of the linker region in the UIM motifs was clearly addressed and a possible interaction mode between the two tandem UIMs of AT3 and Ub or Ub chains was also proposed and discussed in detail.

## Results

### Solution Structures of the Free and Ub-Bound Forms of Tandem AT3-UIM12

**Figure 2:**
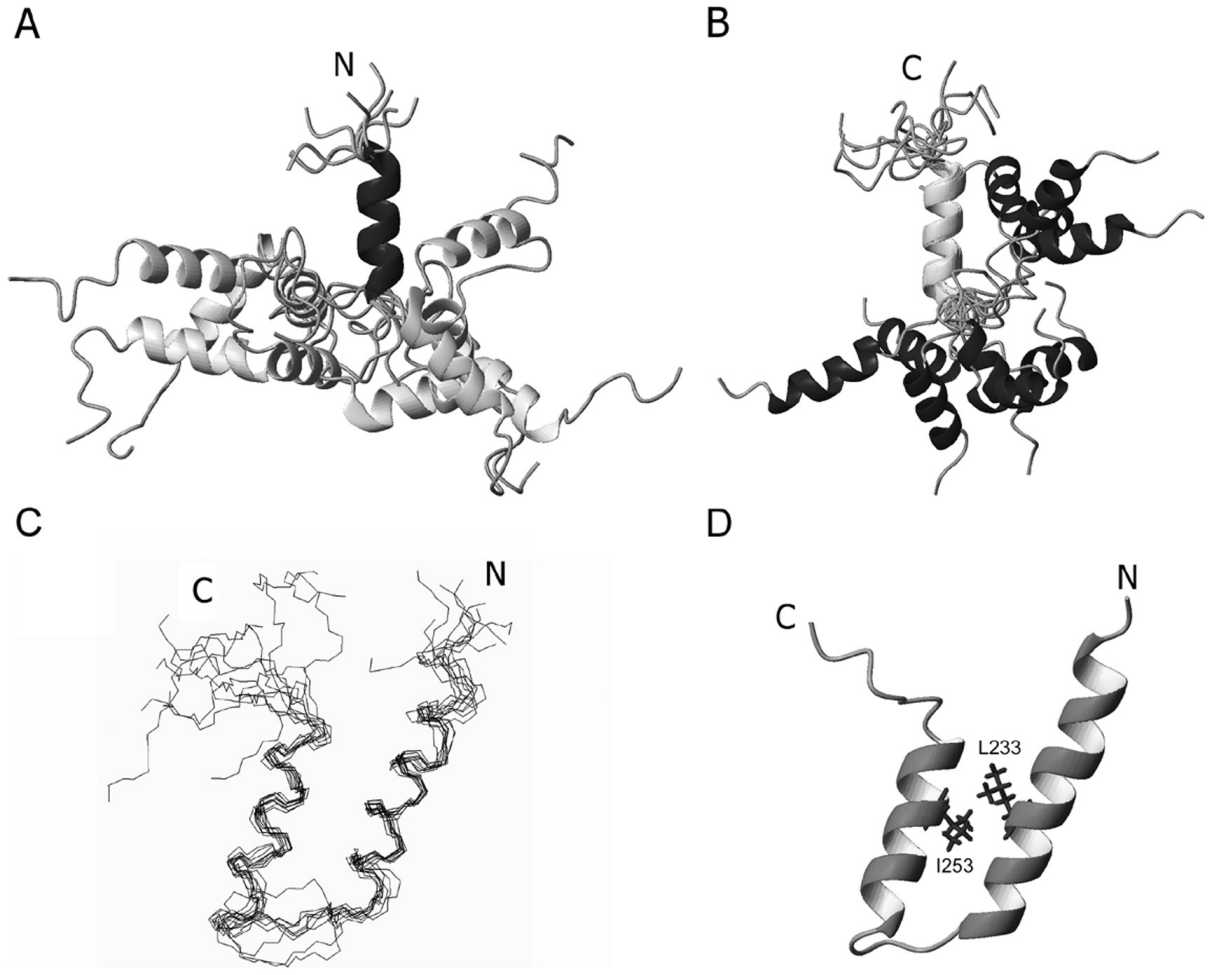
Solution Structures of Tandem AT3-UIM12 in Free and Ub-Bound Forms. A, Ensemble of 10 structures superimposed on the polypeptide backbones of the α-helix of UIM1. B, Ensemble of 10 structures superimposed on those of UIM2. The helical portions of AT3-UIM12 show relative high definition, but the linker region is unstructured and flexible. No tertiary contacts between helices are observed. C, Backbone superposition of 10 lowest-energy structures of AT3-UIM12 in the Ub-bound form. D, Ribbon representation of AT3-UIM12 structure in the Ub-bound form. The structure of the Ub-bound form includes two α-helices, helix-1 (residues 226–238) and helix-2 (residues 247–256), and a linker loop region (residues 239–244) between them. The figures were generated using MOLMOL.

To obtain the binding properties of the tandem UIM motifs with Ub, we firstly assigned the chemical shifts (Supplemental Fig. S1A) and NOEs of AT3-UIM12, and solved its solution structure by heteronuclear multidimensional NMR techniques. A summary of the NMR-derived conformational restraints for structure calculation and the structure model statistics is presented in Supplemental Table S1. Similar to the structures of other tandem UIMs [16], [18], the overall structure of AT3-UIM12 is poorly defined due to relative flexibility of the linker, but the two individual UIM regions are well structured (Fig. 2). We depict an ensemble of 10 lowest-energy NMR structures superimposed on the polypeptide backbones of helix-1 (Fig. 2A) and helix-2 (Fig. 2B), respectively. As expected from the sequence, the structure of AT3-UIM12 is comprised of two α-helices and a flexible linker, ^239^EIDMED^244^. Due to the absence of long-range NOEs (Supplemental Table S1), the tertiary interactions between the two helices are also absent; hence, the orientation of the two helices is less restricted. Thus, the short linker between the two UIM motifs has little contribution to the overall structure definition. Due to the flexibility of the linker region, the average root-mean-square deviations (RMSDs) for the ten structures for backbone and all heavy atoms are relatively high, though the two helical regions have reasonable RMSD values (Supplemental Table S1). The relative independence of the helical orientation provides a large interface for each helical motif to bind with the globular Ub molecule individually; and one AT3-UIM12 can potentially bind with two Ub molecules.

To understand the binding mode of AT3-UIM12, we elucidated the structure of AT3-UIM12 complexed with Ub in solution. Upon binding with Ub, the chemical shifts of AT3-UIM12 change significantly to a higher dispersion (Supplemental Fig. S1B). We assigned the chemical shifts (BioMagResBank database accession number 16405) and the intra-molecular NOEs, and solved the solution structure of AT3-UIM12 in complex with Ub (Supplemental Table S1) (Protein Data Bank accession code 2KLZ). To our surprise, the Ub-bound AT3-UIM12 displays a compact structure with typical helix-loop-helix folding pattern (Fig. 2C and 2D). There are hydrophobic contacts between the two α-helices, for example, Leu233 in helix-1 with Ile253 in helix-2. Compared with the free form that has a flexible linker (RMSD of 1.36), the Ub-bound form becomes relatively rigid with a marginally acceptable RMSD of 0.72. Three residues (Ser236, Arg237 and Gln238) contribute to formation of the longer helix-1, whereas two residues (Glu245, Glu246) are not involved in helix-2. This suggests that Ub binding causes allosteric effects on the structure of AT3-UIM12.

### Backbone Dynamics of AT3-UIM12 Complexed with Ub

**Figure 3:**
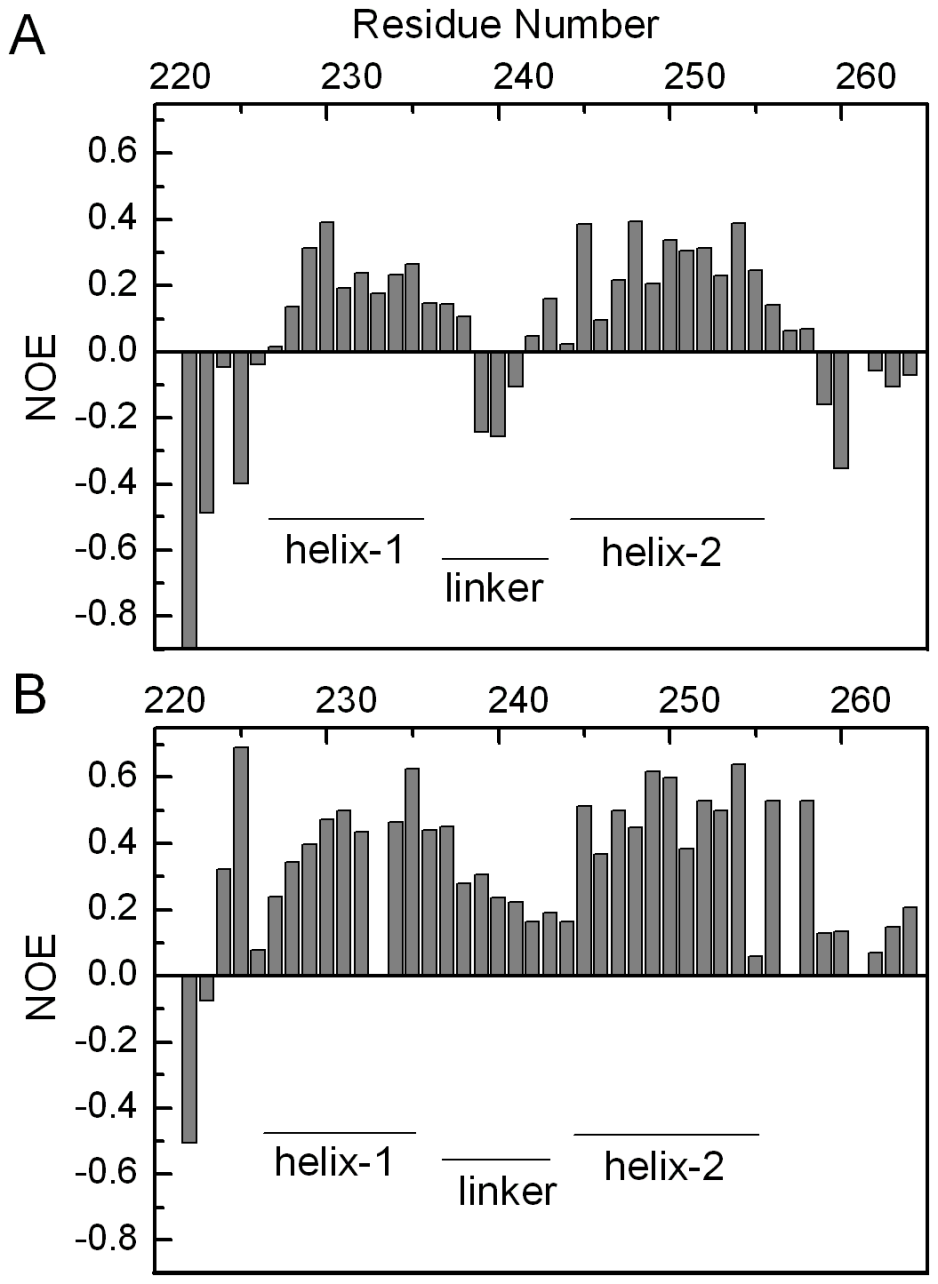
Backbone Dynamics of AT3-UIM12 in the Free and Ub-Bound Forms. A, The ^15^N-{^1^H}-NOE values of backbone amides are plotted against the residue number of AT3-UIM12 in the absence Ub. B, The ^15^N-{^1^H}-NOE values in the presence of two-fold amount of Ub. The secondary structures of AT3-UIM12 are indicated as narrow bars.

The structural analysis suggests that the Ub binding induces conformational arrangement of AT3-UIM12 to a more rigid structure, especially in the linker region. To further ascertain the intrinsic Ub-binding properties, we compared the backbone dynamics of AT3-UIM12 in the presence and absence of Ub. The ^15^N-{^1^H}-NOEs of the backbone amides of AT3-UIM12 in the free and Ub-bound forms were measured (Supplemental Fig. S2), and then the relative NOE intensities for the backbone amides in both forms were delineated versus amino acid sequence of AT3-UIM12 (Fig. 3).

In general, the backbone amides with large ^15^N-{^1^H}-NOE values (0.7–1.0) are expected to have lower flexibilities and to be located in the rigid region, while those with small NOE values (<0.6) are considered to be located at the loops or linker regions that have high flexibility [30]. Due to no defined tertiary structure in the free form of AT3-UIM12, the entire NOE values are relatively low as compared with those in well-folded proteins. The residues in the two helical regions (226–235, 245–256) have hetero-nuclear NOE values of 0.2∼0.4, whereas the linker residues (Glu239, Ile240 and Asp241) give negative NOEs (Fig. 3A). The negative NOEs indicate that this region is much more flexible than other regions. Remarkably, these linker residues in the complex display positive NOE values (∼0.2) (Fig. 3B), suggesting that the internal motions of the linker region get restricted when bound with Ub. At the same time, the two helical regions also exhibit intensity enhancements, thus it is probably that the entire molecule becomes more rigid. Based on these observations, we propose that the two UIM motifs can move freely with diverse orientations in the free form, but upon Ub binding, the linker as well as the two UIM helices become rigid in the complex [31]. Formation of the relatively orientated UIM1 and UIM2 upon Ub binding thus improve the affinity of AT3-UIM12 with Ub efficiently.

### Interactions of Tandem UIM12 and Individual UIMs with Ub

**Figure 4:**
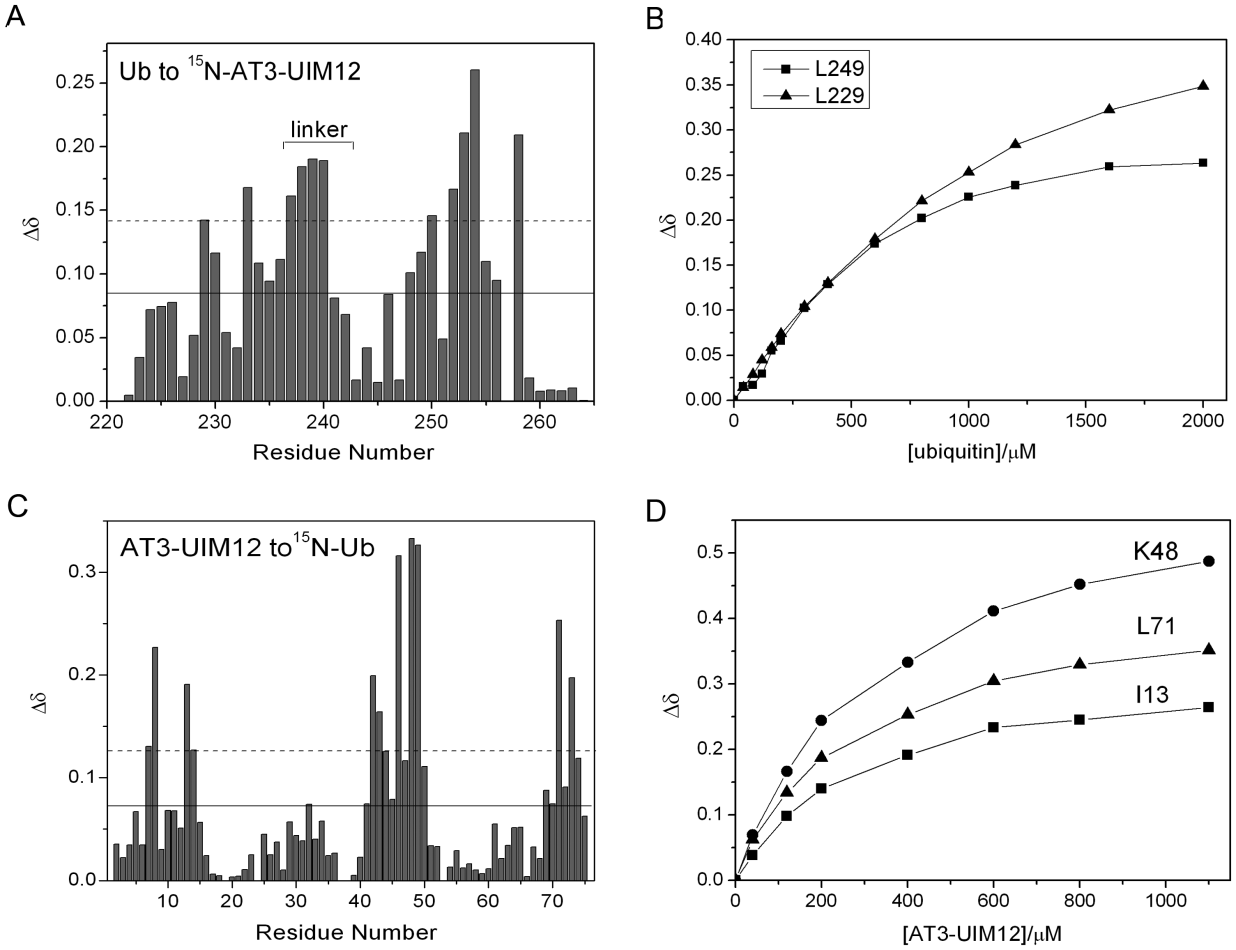
Interaction of Tandem AT3-UIM12 with Ub. A, Diagram of the chemical shift changes (Δδ) of AT3-UIM12 against residue number at an AT3-UIM12/Ub molar ratio of 1∶2. B, Titration curves for two equivalent residues (Leu229 in UIM1 and Leu249 in UIM2) in tandem AT3-UIM12 upon Ub binding. Note that the Δδ value of Leu249 in UIM2 upon Ub titration reaches the plateau with lower amount of Ub than the corresponding Leu229 in UIM1. C, Diagram of the chemical shift changes (Δδ) of Ub against residue number at an Ub/AT3-UIM12 molar ratio of 1∶2. D, Titration curves for three residues (Ile13, Lys48 and Leu71) in Ub upon GB1-tagged AT3-UIM12 binding. The dissociation constant for the binding of AT3-UIM12 with Ub is listed in Table 1. The solid and dashed lines (A & C) indicate the threshold values of mean and mean plus SD for the chemical shift changes.

We also investigated the interaction between the tandem UIM motifs and Ub by GST pull-down assay. The result shows that AT3-UIM12 can bind with GST-Ub, while the third UIM motif (AT3-UIM3) cannot (Supplemental Fig. S3). To map the binding interfaces between AT3-UIM12 and Ub, we collected a series of ^1^H-^15^N HSQC spectra on the ^15^N-labeled AT3-UIM12 in the presence of increasing amount of Ub (Supplemental Fig. S4A). A reciprocal experiment was also carried out on ^15^N-labeled Ub titrated with AT3-UIM12. The profile for the amide chemical shift changes (Δδ) of ^15^N AT3-UIM12 upon titration of Ub at a molar ratio of 1∶2 is shown in Fig. 4A. Surprisingly, besides the conserved residues in the two helices, such as Leu229 and Leu249, Ala232 and Ala252, and Ser236 and Ser256, the chemical shifts of the linker residues (Gln238, Glu239 and Ile240) are also strongly perturbed. The large chemical shift changes imply that the linker region experiences structural and dynamics change or residue contacts upon Ub binding.

The Δδ values upon titration of Ub were extracted from the HSQC spectra of at different molar ratios of AT3-UIM12 to Ub. The chemical shift changes for the equivalent residues, Leu229 in UIM1 and Leu249 in UIM2, respectively, exhibit different saturation curves as Ub titration proceeds (Fig. 4B). Similar results are also observed in Ser236 and Ser256, as well as in Ala232 and Ala252 (Supplemental Fig. S4B). The Δδ values of the residues in UIM2 upon Ub binding reach the plateau with lower amount of Ub than the corresponding ones in UIM1, suggesting that the two UIM motifs bind Ub with different affinities. It is likely that, in AT3-UIM12, the UIM2 moiety may have a higher Ub-binding affinity than UIM1 (Fig. 4B and Supplemental Fig. S4B). Similarly, titration of ^15^N-labeled Ub with AT3-UIM12 causes significant but selective perturbations on the Ub spectra (Fig. 4C). Ub specifically binds with AT3-UIM12 through its typical hydrophobic patch mainly comprised of Leu8, Ile44 and Val70. We have determined the apparent dissociation constant (*K*_D,app_) from the three strongly perturbed residues, Ile13, Lys48 and Leu71 (Fig. 4D). The apparent *K*_D_ value is 97.0±16.9 µM for AT3-UIM12 binding with Ub (Table 1).

**Table 1:** Apparent Dissociation Constants for the Ub Binding Affinities of Tandem AT3-UIM12, Single AT3-UIM Motifs and Their Mutants.

UIM Peptide	*K*_D,app_ (µM)^a^
AT3-UIM12 (WT)	97.0±16.9^b^
AT3-UIM1	215±32
AT3-UIM2	309±30
AT3-UIM3	> 3000
AT3-UIM12-I240A	141±9
AT3-UIM12-I240P	175±3
AT3-UIM12-Q238E/E239T	333±23
GS-Sub^c^	195±17
GS-Ins	16.8±8.3
RAP80-Linker	39.0±11.5

a The dissociation constants were determined from NMR titration of different GB1-tagged UIM peptides to ^15^N-labeled Ub. For comparison, we calculated the apparent *K*_D,app_ values for AT3-UIM12, provided that the two UIM motifs bind Ub equally.

b The chemical shift changes of three individual residues I13, K48 and L71 on Ub were used to calculate the dissociation constants. Data are presented as mean ± SD.

c The sequences of the linker regions for wild-type AT3-UIM12 and its mutants. WT: ^239^EIDMED^244^; GS-Sub: GSSGGS; GS-Ins: EIDGSSGGSMED; RAP80-Linker: EAREVNNQEEK.

In AT3-UIM12, both UIM1 and UIM2 moieties can bind to Ub individually but with different affinities. It is reasonable to raise such a question as why AT3 adopts tandem UIM motifs. Actually, UIM3 is relatively highly conserved in sequence as compared with UIM1 and UIM2, but the pull-down experiment suggests that it is unlikely to bind with Ub (Supplemental Fig. S3). From NMR titration experiments, we calculated the dissociation constants for the three single UIM motifs (Table 1). As expected, the single UIM1 and UIM2 bind Ub much weaker than AT3-UIM12. Different from the situation of UIM moieties in AT3-UIM12 where UIM2 binds Ub a little stronger than UIM1, these two individual UIMs exhibit almost similar binding affinities. This is possibly the result of association of the two UIM motifs linked by the relatively flexible region and the structural transformation of AT3-UIM12 especially in the UIM2 moiety that favors Ub binding. Thus, the tandem form of UIM motifs improves the binding affinities with Ub as compared with single isolated UIMs.

To investigate the linker region that may be critical to the Ub binding, we constructed three mutants of AT3-UIM12 with different linkers (Fig. 1C). Substitution of the linker region (GS-Sub) weakens the binding with Ub considerably, whereas both insertion (GS-Ins) and substitution of the linker region with RAP80 linker (RAP80-Linker) significantly increase its binding affinity with Ub (Table 1). This result indicates that the relatively long linker is beneficial to the Ub binding, possibly due to the increased flexibility of the long linker or formation of a longer α-helix as in RAP80 [19].

### The Hydrophobic Residues of AT3-UIM12 Play Dominant Roles in Ub Binding

**Figure 5:**
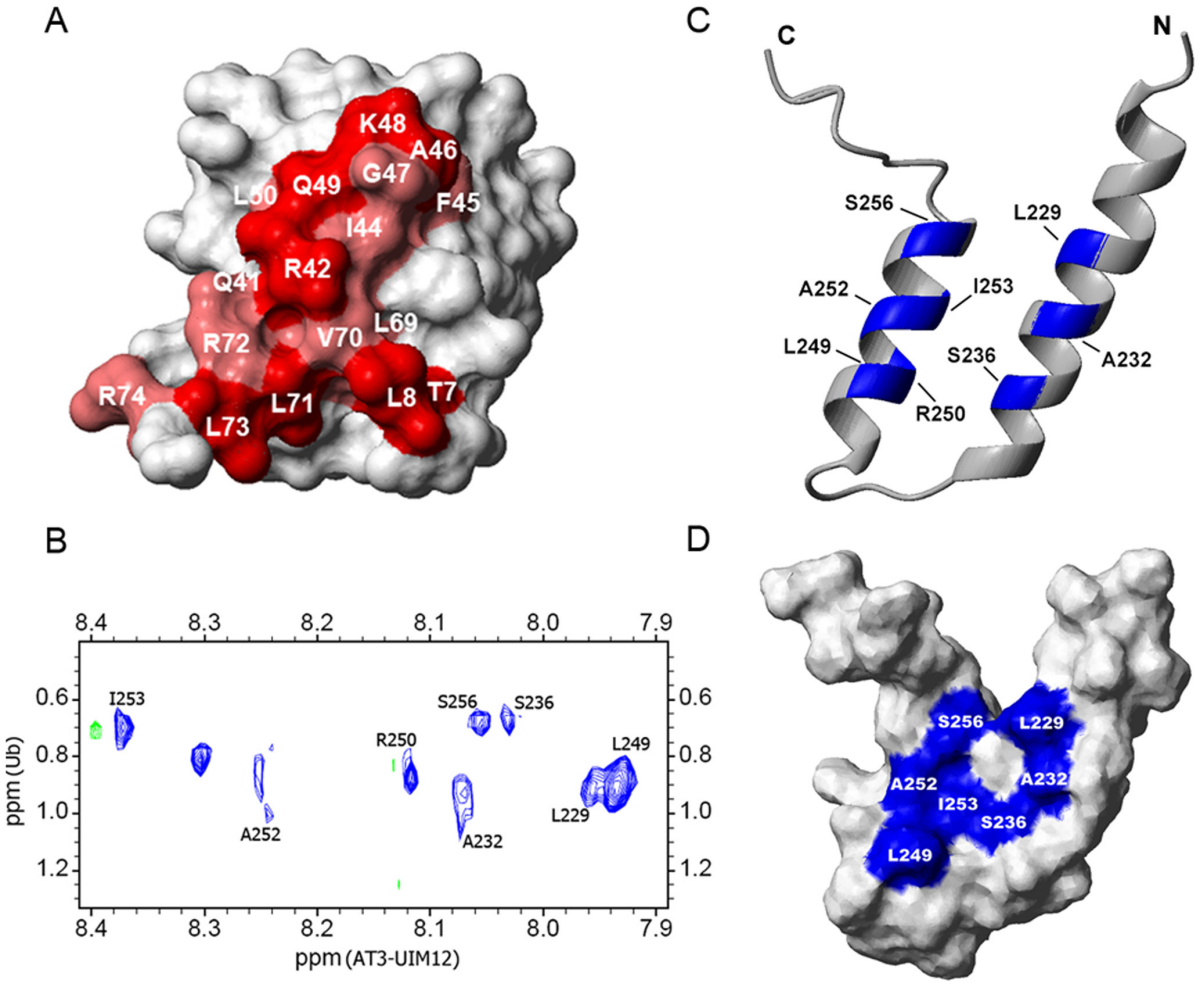
AT3-UIM12 Binds with Ub Through Hydrophobic Interactions. A, Mapping of the significantly perturbed residues by AT3-UIM12 interaction on Ub surface. The residues with chemical shift changes larger than the average have been labeled in pink and those larger than the average plus SD in red (ref. Fig. 3C). The Ub structure is referenced from the crystal structure (PDB code: 1UBQ). B, Intermolecular NOEs observed in the AT3-UIM12-Ub complex. A 1∶2 molar ratio of labeled AT3-UIM12 to unlabeled Ub were mixed for 3D ^13^C-filtered/^15^N-edited NOESY spectrum. The figure shows the NOE peaks projected onto the 2D plane. C, Mapping of the residues that have the intermolecular NOEs with Ub on the ribbon diagram of AT3-UIM12. D, Mapping of the residues that have the intermolecular NOEs with Ub on the surface diagram of AT3-UIM12, showing two patches for binding with two Ub molecules. The ribbon and surface representations were generated by MOLMOL.

Ub interacts with some Ub-binding domains (such as UBA) mainly through its hydrophobic patch composed of Leu8-Ile44-Val70 [32]. In this study, NMR titration of Ub with AT3-UIM12 has given similar information of the binding surface on the Ub structure [33] centered by Leu8, Ile44 and Val70 (Fig. 5A). This canonical binding surface has been reported in several related literatures [18], [34], [35], [36], [37].

We also obtained direct evidence for the specific binding sites on AT3-UIM12 from intermolecular NOEs. Based on the filtered/edited-NOESY spectra recorded on the AT3-UIM12/Ub complex (at a molar ratio of 1∶2), several intermolecular NOE peaks were unambiguously identified. We found that eight residues of AT3-UIM12 have direct contacts with Ub (Fig. 5B). Among these residues interacting with Ub, Leu229, Ala232 and Ser236 are located in the first UIM motif, while their equivalent residues Leu249, Ala252 and Ser256 plus two other residues Arg250 and Ile253 are in the second UIM (Fig. 5C). Definitely, hydrophobic interactions are dominant in the UIM12-Ub complex, for the Leu, Ala and Ile residues constitute the hydrophobic surface on AT3-UIM12. Ser236 and Ser256 may participate in the binding through hydrogen bonds with the backbone of Gly47 in Ub [18]. There are two similar patches but opposite directions on the binding surfaces of AT3-UIM12, each corresponding to one UIM motif and binding with one Ub molecule (Fig. 5D).

Interestingly, there is no intermolecular NOE peak observed for the linker residues Gln238, Glu239 and Ile240, suggesting that these residues are not directly involved in the specific binding. Mutation experiments (I240P and I240A) indicate that these residues have limited effect on the binding affinity. However, the double mutant (Q238E/E239T) of AT3-UIM12 gives a similar binding affinity with individual UIMs, suggesting that double mutation of the linker abolishes the association between the two UIMs (Table 1 and Supplemental Fig. S5). Hence, we presume that the chemical shift perturbations observed in the titration experiments (Fig. 4A) might be caused by local conformational change of AT3-UIM12 upon Ub binding.

### Interactions of AT3-UIM12 with diUb or polyUb Chains

**Figure 6:**
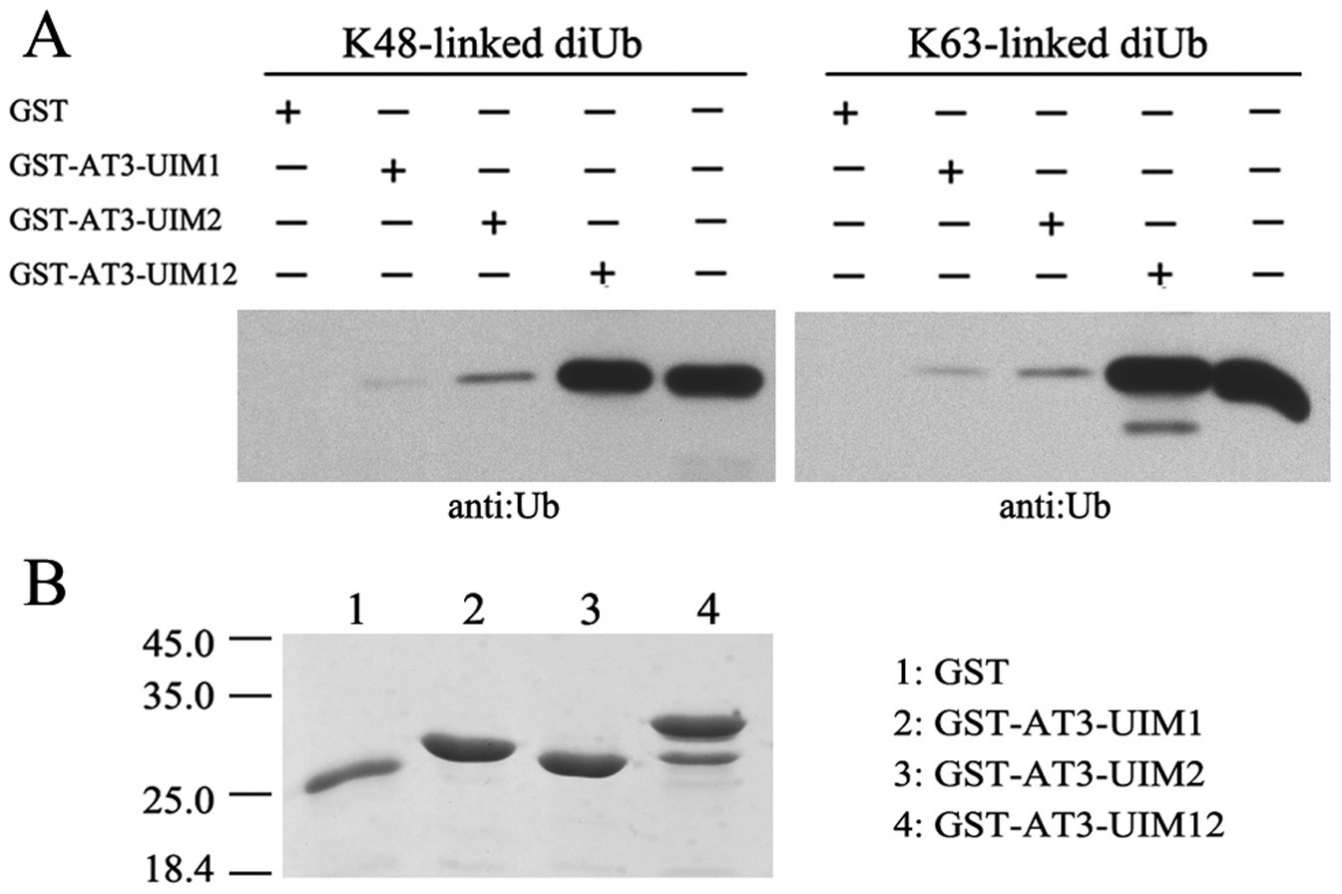
The Cooperative Interaction of AT3-UIM12 with K48- and K63- Linked diUb Chains. A, Pull-down of K48- and K63-linked diUb by GST-fused UIM1, UIM2 or UIM12. The pull-down samples were detected by Western blotting with an anti-Ub antibody. B, SDS-PAGE of the GST-fused proteins (AT3-UIM1, AT3-UIM2 and AT3-UIM12) detected by Coomassie staining.

Since the NMR investigations provided evidence that the tandem AT3-UIM12 binds with monoUb more efficiently than single UIMs, we further examined the binding properties of single UIMs and tandem UIM12 with diUb or polyUb by GST pull-down experiments. The data show that AT3-UIM12 can pull down both K48- and K63-linked diUb more efficiently than the single UIM1 or UIM2 (Fig. 6). Moreover, AT3-UIM12 can bind with the polyUb mixture more strongly than the single UIM1 or UIM2, no matter whether the polyUb chains are linked by K48 or K63 residue (Supplemental Fig. S6). On the other hand, AT3-UIM12 binds with longer Ub chains more efficiently, which is consistent with the previous reports that AT3 preferentially binds with longer Ub chains [38], [39].

**Figure 7:**
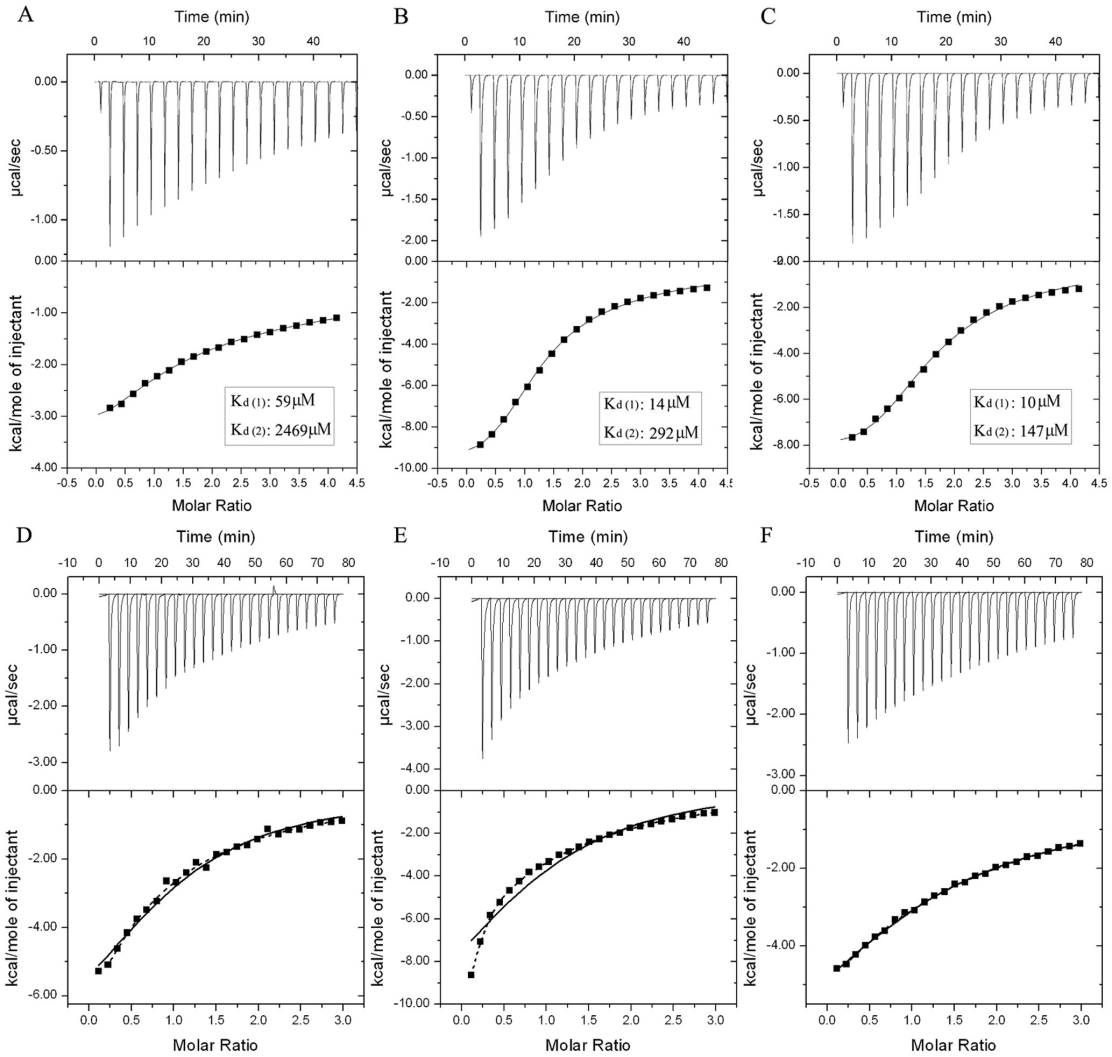
Isothermal Titration Calorimetry Experiments for the Interactions of various AT3-UIM12 forms with diUb types. A, B, C, Titration of different types of diUb with GB1-AT3-UIM12. A, Linear diUb; B, K48-linked diUb; C, K63-linked diUb. By fitting the data with the sequential binding model, these titrations are well-fitted to give two distinct binding affinities. D, E, F, Titration of K48-linked diUb with different AT3-UIM12 forms. D, GS-Sub; E, GS-Ins; F, RAP80-Linker. The concentration of K48-linked diUb is 100 µM and those of the AT3-UIM12 stocks are 2 mM. The titrations are well-fitted to a usual two-site binding model (dashed line) rather than the sequential binding model (solid line). The concentrations of diUb types are 100 µM and those of AT3-UIM12 stocks are 2 mM. The GB1 fusion was used for peptide expression and quantification.

To further quantify the binding affinities of AT3-UIM12 with various types of diUb, we performed ITC and NMR experiments. The data for each ITC titration are well-fitted to a sequential two-site binding model rather than a simple one-site or two-site mode (Fig. 7A-C and Supplemental Table S2). Each titration gives two distinct dissociation constants, suggesting that the binding includes two sites on different AT3-UIM12 conformers, one is weak and the other is relatively strong. The results also indicate that the K48- and K63-linked diUb (*K*_D1_ = ∼10 µM, *K*_D2_ = 100∼300 µM) bind AT3-UIM12 with similar affinities, but much more strongly than the linear diUb (*K*_D1_ = 59 µM, *K*_D2_ = 2469 µM). This implies that the strong site on AT3-UIM12 may originate from the allosteric effect during diUb binding. Similar results are also obtained from NMR titrations of AT3-UIM12 with different diUb types, respectively (Supplemental Fig. S7). The binding of AT3-UIM12 with K48-linked diUb is relatively strong, then with linear diUb, and that with monoUb is weak. This suggests that AT3-UIM12 binds different diUb types with different orientations, adaptable to the isopeptide-linked types but inadaptable to the linear one.

We also determined the binding affinities of the three linker variants with K48-linked diUb by ITC (Fig. 7D-F and Supplemental Table S2). As expected, the three mutants of AT3-UIM12 in linker region bind with diUb stronger than with monoUb. Different from those for wild-type AT3-UIM12 (Fig. 7B), the titration data for GS-Sub (Fig. 7D) and GS-Ins (Fig. 7E) cannot be well-fitted to a sequential two-site binding model but can be fitted to a conventional two-site binding model, indicating that alteration of the linker may interrupt the association of two UIM helices binding with diUb. This suggests that the linker region in AT3-UIM12 plays an important role in the association of the two UIM helices. Thus, our biochemical studies reveal that the binding affinity of tandem AT3-UIM12 for polyUb is far larger than those of the individual UIMs, and the linker region in AT3-UIM12 is conducive to structural transformation that may be preferential for the configuration of isopeptide-linked diUb.

## Discussion

More and more multiple copies of domain or motif are found in functional proteins, like the tandem WW domain in HYPA/FBP11 [40], [41], the double SH3 domain in p47phox [31], the two UbL domains in ISG15 [42], [43], and the tandem UBA or UIM domains in several proteins related to Ub-proteasome system [3], [32], [44]. The presence of multiple ligand-binding sites within a protein affords opportunities for multiple simultaneous interactions, and is then able to significantly enhance or reduce the efficiency of ligand binding by an allosteric and cooperative (positively or negatively) manner, as compared with the binding to a single domain or motif.

As to the multi-UIM-containing proteins, the mutations within a single UIM may produce larger effects than expected if these UIMs function in a non-redundant fashion [5], [7]. Furthermore, the relatively modest affinity of single UIM for monoUb leaves open the possibility that the mechanism of high-affinity binding may involve cooperation between multiple UIMs [14], [15], [45] and polyUb chains [46], [47]. However, the previous studies on tandem UIMs from S5a and Vps27p did not find any obvious cooperation for Ub binding [16], [17], [18]. The UIM portions in both proteins form typical α-helices and interact with Ub directly and individually. On the other hand, some tandem motifs or domains, though non-cooperatively, can mediate formation of a complex or oligomer by binding with polyUb [47], [48]. The S5a protein includes another short α-helix in the linker region, however, this helix does not participate in the Ub binding and binding cooperation [18]. The linker region of Vps27p is random and non-structural, and has also been proved not to contribute to the interactions between UIMs and Ub [16]. The interactions of the two UIMs in S5a and Vps27p with Ub have been thought to be independent and non-cooperative. These observations thus argue against a cooperative binding mechanism in the tandem UIMs binding with Ub.

The sequence alignment of several tandem UIMs (Fig. 1B) displays high homology in UIM motifs from diverse proteins, but the sequences and lengths of the linker regions between two UIMs are largely different. There are about 20∼50 residues inserted in between two UIMs of S5a and Vps27, but the linker region between the two UIMs of AT3 is very short. These adjoining UIM motifs in AT3 raise a possible cooperativity for binding with Ub. Our structure determination and titration experiments reveal that the individual UIMs of AT3 can bind Ub respectively but with lower affinities. The *K*_D_ values for AT3-UIM1 (215 µM) and AT3-UIM2 (309 µM) are comparable with those for S5a (∼350 µM for UIM1 and 73 µM for UIM2) and Vps27p (277 µM for UIM1 and 117 µM for UIM2). When the two UIMs are juxtaposed to interact with Ub, the binding affinity of tandem UIM12, especially the UIM2 moiety, is increased obviously with an apparent *K*_D_ value of 97.0 µM. As to the diUb or polyUb binding, the binding affinity of AT3-UIM12 with polyUb is dramatically increased when UIM1 and UIM2 are linked together (Fig. 6 and Supplemental Fig. S6). It seems that the cooperative interaction of AT3-UIM12 with diUb or polyUb is more remarkable than that with monoUb (Supplemental Fig. S7). Although extending the linker significantly increases the binding affinity of AT3-UIM12 with monoUb (Table 1), it may abolish its cooperative interaction with diUb (Fig. 7). Therefore, the altered affinities of AT3-UIM12 for monoUb or diUb may result from structural transformation of the linker region. The backbone dynamics provides further evidence for the conformational change in the linker region, which possibly allows cooperative interaction with Ub.

**Figure 8:**
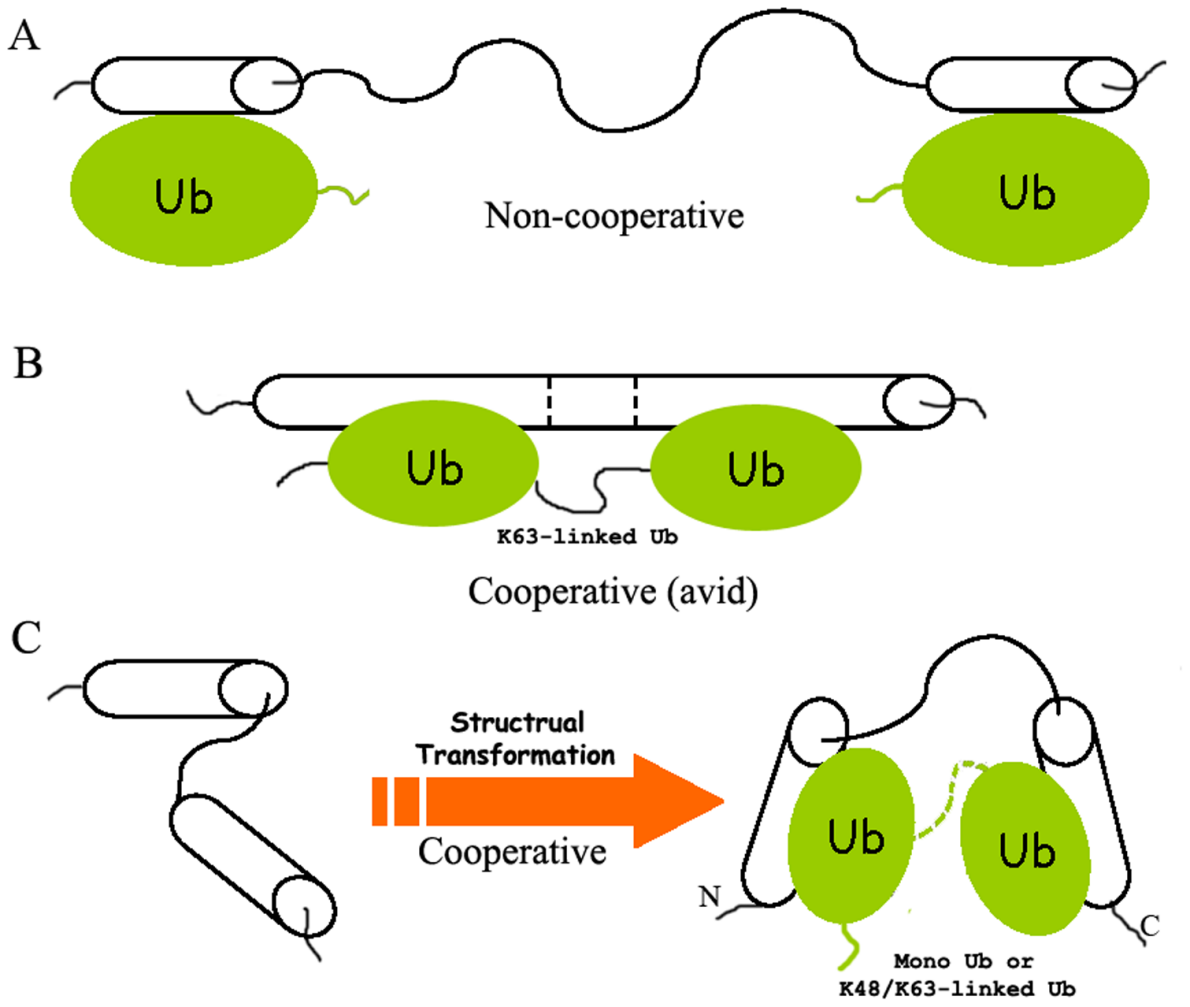
Schematic Representation of the Tandem UIM Motifs with Various Forms of Ub Chains. A, Non-cooperative binding mode. A representative for the mode is the tandem UIM motifs in Vps27p [16], which bind with Ub independently and non-cooperatively. B, Avid binding mode. The tandem UIM motifs in RAP80 bind with K63-linked diUb by forming a long α-helical structure [19]. The predicted linker becomes rather rigid and may be preferable to the configuration of K63-linked diUb. C, Cooperative binding mode. A short linker region (such as in AT3-UIM12) tends to be involved in the cooperative binding with Ub chains or ubiquitinated substrates. This cooperation is also likely to be occurred in poly- or multiUb. When the tandem UIM motifs bind with any form of Ub, a conformational arrangement and dynamics change occur in the linker region that may favor Ub binding. The dashed line indicates that either mono- or diUb is involved in the binding mode, and Ubi denotes different forms of Ub, standing for mono-, di-, poly- or multiUb.

The length of the linker region in tandem UIM motifs is important for a protein to bind with Ub chains, including mono-, poly- and multiUb. In S5a and Vps27p, the long loop linker affords them to bind with any types of Ub but generally weakly and non-cooperatively. The two or three helical portions in the tandem UIM motifs have their respective orientations; thus they bind Ub with different directions. Binding of the individual UIM motif with Ub has no relation to each other and is independently and non-cooperatively (Fig. 8A). Interestingly, although RAP80 contains a rather short linker (7 residues as referred and ∼11 as aligned in Fig. 1), the two UIM helices merge together with the linker into a continuous long α-helix when bound with K63-linked diUb [19], [46]. Each helical portion binds independently to a Ub unit of K63-linked diUb (Fig. 8B). This binding mode, though efficient and specific for K63-linked diUb (K63-specific avidity), may be not adaptable to the binding of K48-linked or linear diUb, due to the steric hindrance. Because the long helical linker is rather rigid during ligand binding, the tandem UIMs of RAP80 can only specifically bind to K63-linked diUb. However, in AT3, although the two UIM helical portions are less orientated in solution due to the flexible linker (Fig. 2A & 2B), they become relatively compact and orientated when the UIM motifs bind with Ub (Figure 2C & 2D). Meanwhile, the linker region is more ordered in the complex form (Fig. 3). These two oriented helices in the Ub-bound form are likely beneficial to the binding with diUb, polyUb or ubiquitinated substrates, no matter whether K48- or K63-linked (Fig. 8C). This cooperation mode is also observed in the tandem WW domains of HYPA/FBP11 binding with N- and C-portions of proline-rich region in huntingtin (Htt) [41], and the tandem PHD and bromodomains in sumoylation and gene regulation [49]. Sterically, in this binding mode, the configuration of two Ub units in diUb is presumed to match the orientation of the helical orientation of AT3-UIM12. Thus, we propose that the cooperation is not only crucial but also beneficial to the binding of AT3 protein with polyUb or multiUb, and to the function as a deubiquitinating enzyme.

The *in vitro* binding data demonstrated striking differences between UIMs in their protein-protein interactions despite their small size and highly conserved core residues. Recent work investigated the inhibiting activity of UIM to the aggregation of Htt, and demonstrated that different UIM has different effect on the polyQ-expanded aggregates [50]. The UIMs of AT3, S5a, Eps15 and Hrs could be recruited to the aggregate, whereas the UIMs of epsin, HSJ1 and USP25 did not co-localize with Htt-65Q-CFP. Only the expression of AT3 and S5a UIMs can decrease aggregation of Htt-65Q, but they have distinct effects on the aggregate formation. In the case of AT3, the third UIM, along with UIM1 and UIM2, works in combination for reducing aggregate formation [50]. Since the isolated single UIMs might serve as potential inhibitors of polyQ aggregation *in vivo*, the detailed investigation into UIM-Ub interaction will be helpful to understand the mechanism.

In summary, we have solved the solution structures of AT3-UIM12 in both free- and Ub-bound forms and elucidated its binding properties with various Ub chains including monoUb, diUb and polyUb. Both AT3-UIM12 and the single UIM motifs can interact with the hydrophobic patch of Ub through its conserved hydrophobic residues. The tandem motifs show higher binding affinity for mono- or polyUb than any individual UIMs. Also, AT3-UIM12 may experience a structural transformation and dynamics change during Ub binding. The linker region does not participate in the direct binding, but the chain rigidity significantly increases when it binds to Ub. Taken together, we propose that the tandem UIM motifs in AT3 interacting with Ub chains undergo a cooperative manner that may modulate its binding efficiency with monoUb, polyUb or ubiquitinated substrates, which may probably be beneficial to selectively regulating the function of AT3 protein in cell.

## Materials and Methods

### Construction of the Plasmids

The plasmid pPROEX HTa-ataxin-3 was kindly provided by Peter Breuer at Max-Planck-Institute for Biochemistry. All the subclones of tandem AT3-UIM12 (residues 222–263) and its linker mutants (GS-Sub, GS-Ins, RAP80-Linker), and single UIM motifs, AT3-UIM1 (residues 222–241), AT3-UIM2 (residues 242–261) and AT3-UIM3 (residues 335–354) were constructed via PCR method with the above AT3 as template. The PCR products were ligated into the pGBTNH or pGEX-4T vector with *BamH* I and *Xho* I restriction sites to produce GB1-fused or GST-fused UIM proteins [51]. The mutants of AT3-UIM12 (I240A, I240P and Q238E/E239T) were prepared using PCR site-directed mutagenesis technique. The cDNA encoding Ub was cloned into pET-3a plasmid using the *Nde* I/*BamH* I sites. All the plasmids were verified by DNA sequencing.

### Protein Expression and Purification

The tandem AT3-UIM12 was overexpressed in *E. coli* BL21 (DE3) strain (Invitrogen). Bacterial cells were grown at 37°C in LB media and the expression was induced by adding IPTG to a final concentration of 0.2 mM. The culture was incubated for an additional 10 h or overnight at 22°C. All GB1-fused UIM proteins were initially purified by a Ni^2+^-NTA column (Qiagen), followed by size-exclusion chromatography (Superdex-75, GE Healthcare). ^15^N- and ^15^N/^13^C-labeled UIM proteins were overexpressed in M9 minimal media using [^15^N]-NH_4_Cl and [^13^C]-glucose as the sole nitrogen and carbon sources (Cambridge Isotope Laboratories). After the GB1-fused AT3-UIM12 was eluted from Ni-NTA column, the GB1 tag was removed by thrombin digestion followed by a second Ni^2+^-NTA column and a Superdex-75 column. The resulting peptide fragments contain two extra residues, Gly and Ser, at the N-terminus and a His_6_ tag at the C-terminus. Purified peptides were dialyzed exhaustively against water, lyophilized, and stored at −20°C.

Bacterial cells harboring pET-3a-Ub plasmid were grown at 37°C in LB or M9 minimal media until an OD_600_ of 0.6 was reached. Ub expression was induced at 22°C for about 10 h by adding IPTG to a final concentration of 0.2 mM. The protein was purified sequentially by acetic acid denaturation, 16/10 SP XL column and 16/60 Superdex-75 column chromatography. ^15^N-labeled Ub was prepared with ^15^NH_4_Cl as the sole nitrogen in M9 media. Purified Ub was dialyzed against water, lyophilized and stored at −20°C. The linear diUb protein was expressed and purified from bacterial cells harboring pET-22b-Ub2 plasmid. The K48- and K63-linked diUb proteins were prepared according to the literatures [52].

### NMR Spectroscopy

^15^N- or ^15^N/^13^C-labeled samples (∼1 mM) were dissolved in a PBS buffer (20 mM phosphate, 100 mM NaCl, pH 6.5) containing 8% or 100% D_2_O and 0.02% (w/v) NaN_3_. A mixture of labeled AT3-UIM12 and unlabeled Ub with a molar ratio of 1∶4 was used for recording the spectra of Ub-bound form of AT3-UIM12. All the NMR experiments were performed at 25°C on a Varian UNITY INOVA 600 MHz spectrometer equipped with three RF channels and a triple-resonance pulsed-field gradient probe. The backbone and side-chain ^1^H, ^15^N and ^13^C resonances were assigned based on the spectra of 3D HNHA, HNCO, HNCACB, CBCA(CO)NH, CC(CO)NH, and 3D ^15^N TOCSY-HSQC, ^13^C HCCH-TOCSY. NOE distance restraints for structure calculations were obtained from 3D ^15^N-edited NOESY and ^13^C-edited NOESY (aliphatic ^13^C regions). Intermolecular NOEs between AT3-UIM12 and Ub were obtained from 3D F1 ^13^C/^15^N-filtered-F3 ^15^N-edited NOESY experiments as described [53].

A steady-state heteronuclear ^15^N-{^1^H}-NOE experiment [30] was performed for ^15^N-labeled tandem AT3-UIM12 in the presence and absence of Ub protein. The spectra for measuring the heteronuclear NOEs were recorded with a 2-second relaxation delay, followed by a 3-second period of proton saturation. In the absence of proton saturation, the spectra were recorded by a relaxation delay of 5 s. The NOE data were processed by using NMRPipe software [54] and analyzed by Sparky (T.D. Goddard and D.G. Kneller, Sparky 3, University of California, San Francisco). The steady-state ^15^N-{^1^H}-NOE enhancements were calculated as the ratio of peak intensities in spectra recorded with or without proton saturation.

### Structure Calculation and Analysis

The CNS program [55] with the ARIA module [56] was adopted to assign NOE peaks and to calculate structures. The protein structures were assessed by using PROCHECK [57] and were displayed by the MOLMOL program [58]. Hydrogen bond restraints (two per hydrogen bond) were generated by a combination of H/D exchange data, medium-range NOEs, and chemical shift index. Backbone dihedral angle restraints (φ and ψ) were derived from the TALOS program [59]. For AT3-UIM12, the restraints used for structure calculation are summarized in Table S1. The calculation in combination with iterative NOE peak assignments was performed for 9 cycles, and a total of 200 structures were finally obtained. Ten structures with the lowest energies, which exhibit no NOE violation >0.3 Å and no dihedral violation >5 Å, were selected and displayed.

### NMR Titration Studies

^15^N-Ub at a molar concentration of ∼0.2 mM was titrated with increasing amount of UIM peptides or its mutants. A series of ^1^H-^15^N HSQC spectra of Ub were obtained and the chemical shift changes were measured for each molar ratio. The sequence-specific backbone assignment for Ub was achieved by comparing the almost identical resonance dispersion of Ub on the ^1^H-^15^N HSQC spectra as reported previously [60]. The combined average chemical shift changes (Δδ_ave_) were calculated as Δδ_ave_  = [(0.2*Δδ_N_)^ 2^ + (Δδ_HN_)^ 2^]^1/2^, where Δδ_HN_ and Δδ_N_ are the chemical shift changes in the ^1^H and ^15^N dimensions, respectively. Similarly, the Δδ_ave_ values of ^15^N-AT3-UIM12 titrated by Ub diUb were also obtained. The dissociation constant (*K*_D_) for the UIM-Ub binding was obtained by fitting the titration curves [61]. For the binding of AT3-UIM12 with Ub, an apparent dissociation constant (*K*_D,app_) was obtained by applying a simplified 1∶2 fit, provided that the two UIM motifs bind Ub with equal binding affinity.

### GST Pull-down Assay

For pull-down assay, the GST-fused Ub was added to the glutathione Sepharose 4B beads (Amersham Biosciences) in a PBS buffer (10 mM Na_2_HPO_4_, 140 mM NaCl, 2.7 mM KCl, 1.8 mM KH_2_PO_4_, pH 7.4), and the suspension was agitated at 4°C for 30 min. The beads were washed three times in the same buffer to remove any unbound protein. An equal molar amount of GB1-fused UIM12 or UIM3 of AT3 (containing a C-terminal His_6_-tag) was added, and the suspension was agitated at 4°C for about 2 h. The beads were recovered by centrifugation, and, after excessive washing, the sample was re-suspended in the sample buffer and subjected to SDS-PAGE, followed by Coomassie staining or Western blotting with an anti-His antibody. Similar pull-down experiments were carried out between GST-fused AT3 UIMs and diUb or polyUb. The GST-fused AT3-UIM1, -UIM2 or -UIM12 was incubated for 2 h with either K48-linked or K63-linked diUb (8.5 µg, 0.5 µM) or polyUb mixture (2.5 µg) with a chain length ranging from one to seven (Ub1∼7, purchased from Boston Biochem Inc.) The pull-down samples were subjected to SDS-PAGE, followed by Western blotting with an anti-Ub antibody.

### Isothermal Titration Calorimetry

The ITC experiments were performed on a MicroCal iTC200 at 25°C in an ITC buffer (25 mM Tris-Cl, 50 mM NaCl, pH 7.5). The GB1-AT3-UIM12 protein (2 mM) stocked in a syringe was injected into a 300-µL sample of linear, K48- or K63-linked diUb with the same buffer. The concentration of diUb sample in the cell was 100 µM as determined by spectrophotometry with a coefficient of 2980 cm^−1^M^−1^ at 280 nm. The binding constants were calculated by fitting the data with one-site, two-site or sequential binding model using Origin 7.0 software (OriginLab Corp.) for ITC.

## Supporting Information

Table S1 Experimental Restraints and Structural Statistics of Free and Ub-Bound Forms of AT3-UIM12
(0.08 MB PDF)

Table S2 Summary of the ITC data for AT3-UIM12 binding with diUb chains by different fitting models
(0.01 MB PDF)

Figure S1 1H-15N HSQC Spectra Showing Assignments of Chemical Shifts of the AT3-UIM12 Forms. (A) Free AT3-UIM12 form. The residues are numerated according to the sequence of AT3. (B) Ub-bound AT3-UIM12. The spectrum of Ub-bound form was recorded at the AT3-UIM12/Ub ratio of 1∶4.
(0.12 MB PDF)

Figure S2 Steady-state hetero-nuclear 15N-{1H}-NOE experiments of AT3-UIM12 in the absence (A) and presence (B) of Ub. The spectra on the left were recorded with proton saturation, while those on the right were recorded without proton saturation. The ratios of the peak intensities represents the hetero-nuclear 15N-{1H}-NOE values for the backbone amides. Red, positive NOE; green, negative NOE.
(0.42 MB PDF)

Figure S3 Pull-down Experiment Showing Bindings of AT3-UIM12 and AT3-UIM3 with Ub. The GST pull-down experiments were carried out between GST-Ub and AT3-UIMs, and detected by Coomassie staining and Western blotting with anti-His antibody. AT3-UIM12 pulled down by GST-Ub is indicated by an asterisk. AT3-UIM12 refers to the tandem UIMs of AT3 (residues 222–263); and AT3-UIM3 denotes the third UIM motif (residues 335–354).
(0.08 MB PDF)

Figure S4 NMR Titration Showing the Interaction of Tandem AT3-UIM12 with Ub. (A) Traces for the chemical shift changes of the representative residues of AT3-UIM12 upon Ub titration. The peaks are colored from red (UIM/Ub  = 1∶0) to coral (1∶6). (B) Titration curves for two equivalent residues, Ser236 in UIM1 and Ser256 in UIM2 (left panel), and Ala232 and Ala252 (right), in tandem AT3-UIM12 upon increasing amount of Ub. Note that the Δδ values of the residues in UIM2 moiety upon Ub titration reach the plateau with lower amount of Ub than the corresponding ones in UIM1.
(0.14 MB PDF)

Figure S5 Interaction of the Linker Mutants of AT3-UIM12 with Ub. The titration curves for wild-type AT3-UIM12 and its linker mutants (I240A, I240P, Q238E/E239T) binding with Ub are plotted using the chemical shift changes of Leu71 of Ub as a representative. The dissociation constants for these mutants binding with Ub are listed in Table 1.
(0.03 MB PDF)

Figure S6 Pull-down Experiments Showing the Interactions of Tandem UIM12 and Individual UIMs of AT3 with Different polyUb Chains. The Pull-down experiments were performed on various lengths of polyUb (Ub1∼7, K48- or K63-linked) with GST-fused UIM1, UIM2 or UIM12. The protein bands were detected by Western blotting with an anti-Ub antibody.
(0.13 MB PDF)

Figure S7 Interactions of Tandem AT3-UIM12 with Different Forms of Ub by NMR Titrations. (A) Titration of 15N-labeled AT3-UIM12 with monoUb. (B) Titration of 15N-labeled AT3-UIM12 with linear diUb. (C) Titration of 15N-labeled AT3-UIM12 with K48-linked diUb. With the progress of K48-linked diUb titration, some peaks in the 1H-15N HSQC spectra experience large chemical shift changes and peak broadening. (D) & (E) Titration curves for two representative peaks (Q230 and S236) showing distinct binding affinities for monoUb, linear diUb and K48-linked diUb.
(0.08 MB PDF)
