# Supplementary material for: Structural Transformation of the Tandem Ubiquitin-Interacting Motifs in Ataxin-3 and Their Cooperative Interactions with Ubiquitin Chains
Source: PLoS One. 2010 Oct 7;5(10):e13202. doi: 10.1371/journal.pone.0013202 (PMC2951365; doi:10.1371/journal.pone.0013202)
Supplement: Table S1 — Experimental Restraints and Structural Statistics of Free and Ub-Bound Forms of AT3-UIM12 (0.08 MB PDF) [file pone.0013202.s001.pdf]

Table S1. Experimental Restraints and Structural Statistics of Free and Ub-Bound Forms of AT3-UIM12

	AT3-UIM12 (free)	AT3-UIM12 (Ub-bound)
Number of experimental restraints		
Total unambiguous distance restraints	205	345
Intra residual	113	202
Sequential (| i - j | = 1)	68	95
Medium range (2 ≤ | i – j| ≤ 5)	24<br>0	38<br>6
Long range (| i – j| >5)	28	30
Hydrogen bonds restraints		
Dihedral angle restraints	24	26
ϕ:	24	26
ψ:		
Structure model statistics		
R.m.s.d. from experimental restraints		
NOE distances (Å)	0.016±0.003	0.023±0.003
Dihedral angles (deg.)	0.212±0.172	0.360±0.190
R.m.s.d. from idealized geometry		
Bonds (Å)	0.003±0.000	0.003±0.000
Angles (deg.)	0.480±0.023	0.525±0.003
Impropers (deg.)	1.315±0.149	1.343±0.199
Ramachandran analysis		
Residues in most favored regions (%)	76.7	79.3
Residues in additionally allowed regions (%)	20.0	18.1
Residues in generously allowed regions(%)	1.2	2.1
Residues in disallowed regions(%)	2.1	0.5
Average atomic RMSDs from the mean structure		
All atoms (Å)	7.17	4.07
All atoms except disordered regions (Å)	4.63	1.75
Backbone atoms (N, C_α, C′)		
All residues (Å)	6.44	3.44
All residues excluding disordered regions (Å)	3.21	1.10
Secondary structure elements (Å)		
Helix-1: Residues 226-235 (free), 226-238 (bound)	0.32	0.25
Linker: Residues 236-244 (free), 239-246 (bound)	1.36	0.72
Helix-2: Residues 245-256 (free), 247-256 (bound)	0.33	0.31
