# Supplementary material for: Structural Transformation of the Tandem Ubiquitin-Interacting Motifs in Ataxin-3 and Their Cooperative Interactions with Ubiquitin Chains
Source: PLoS One. 2010 Oct 7;5(10):e13202. doi: 10.1371/journal.pone.0013202 (PMC2951365; doi:10.1371/journal.pone.0013202)
Supplement: Table S2 — Summary of the ITC data for AT3-UIM12 binding with diUb chains by different fitting models (0.01 MB PDF) [file pone.0013202.s002.pdf]

Table S2 Summary of the ITC data for AT3-UIM12 binding with diUb chains by different fitting models

Binding Pair	$K_{d\,(1)}$; $K_{d(2)}$ (M^{-1})	Chi^2/DOF	Fitting model	Evaluation
UIM12/Linear diUb	5.95×10^{-5}; 2.47×10^{-3}	4.1×10^{2}	Sequential [a]	+ [b]
UIM12/K48-diUb	1.43×10^{-5}; 2.92×10^{-4}	6.5×10^{3}	Sequential	+
UIM12/K63-diUb	1.02×10^{-5}; 1.47×10^{-4}	1.1×10^{4}	Sequential	-
GS-Sub/K48-diUb	1.81×10^{-4}; 7.69×10^{-7}	9.4×10^{3}	Conventional	+/-
GS-Ins/K48-diUb	4.85×10^{-6}; 1.10×10^{-4}	1.4×10^{3}	Conventional	+
RAP80L/K48-diUb	9.43×10^{-7}; 2.79×10^{-4}	1.0×10^{3}	Conventional	+

[a] Conventional, conventional two-site binding model; Sequential, sequential two-site binding model.

[b] +, good fitting; +/-, moderate; -, bad fitting.
