# Supplementary material for: Structural Transformation of the Tandem Ubiquitin-Interacting Motifs in Ataxin-3 and Their Cooperative Interactions with Ubiquitin Chains
Source: PLoS One. 2010 Oct 7;5(10):e13202. doi: 10.1371/journal.pone.0013202 (PMC2951365; doi:10.1371/journal.pone.0013202)
Supplement: Figure S1 — 1H-15N HSQC Spectra Showing Assignments of Chemical Shifts of the AT3-UIM12 Forms. (A) Free AT3-UIM12 form. The residues are numerated according to the sequence of AT3. (B) Ub-bound AT3-UIM12. The spectrum of Ub-bound form was recorded at the AT3-UIM12/Ub ratio of 1∶4. (0.12 MB PDF) [file pone.0013202.s003.pdf]

Figure S1

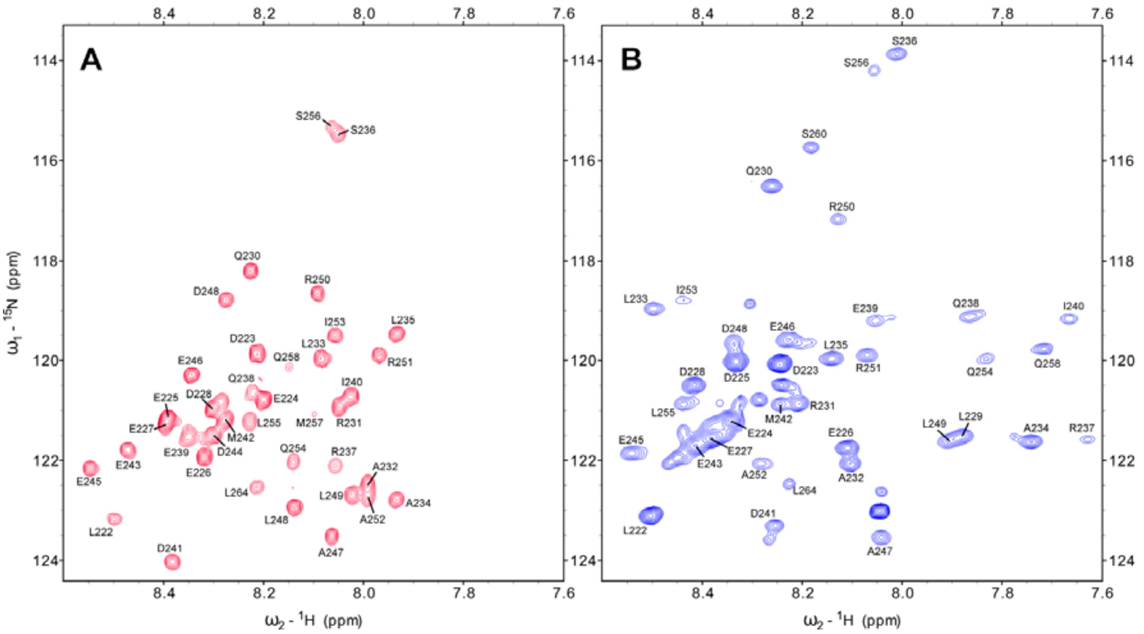

Figure S1. ^{1}H-^{15}N HSQC Spectra Showing Assignments of Chemical Shifts of the AT3-UIM12 Forms. (A) Free AT3-UIM12 form. The residues are numerated according to the sequence of AT3. (B) Ub-bound AT3-UIM12. The spectrum of Ub-bound form was recorded at the AT3-UIM12/Ub ratio of 1:4.
