# Supplementary material for: Structural Transformation of the Tandem Ubiquitin-Interacting Motifs in Ataxin-3 and Their Cooperative Interactions with Ubiquitin Chains
Source: PLoS One. 2010 Oct 7;5(10):e13202. doi: 10.1371/journal.pone.0013202 (PMC2951365; doi:10.1371/journal.pone.0013202)
Supplement: Figure S2 — Steady-state hetero-nuclear 15N-{1H}-NOE experiments of AT3-UIM12 in the absence (A) and presence (B) of Ub. The spectra on the left were recorded with proton saturation, while those on the right were recorded without proton saturation. The ratios of the peak intensities represents the hetero-nuclear 15N-{1H}-NOE values for the backbone amides. Red, positive NOE; green, negative NOE. (0.42 MB PDF) [file pone.0013202.s004.pdf]

Figure S2

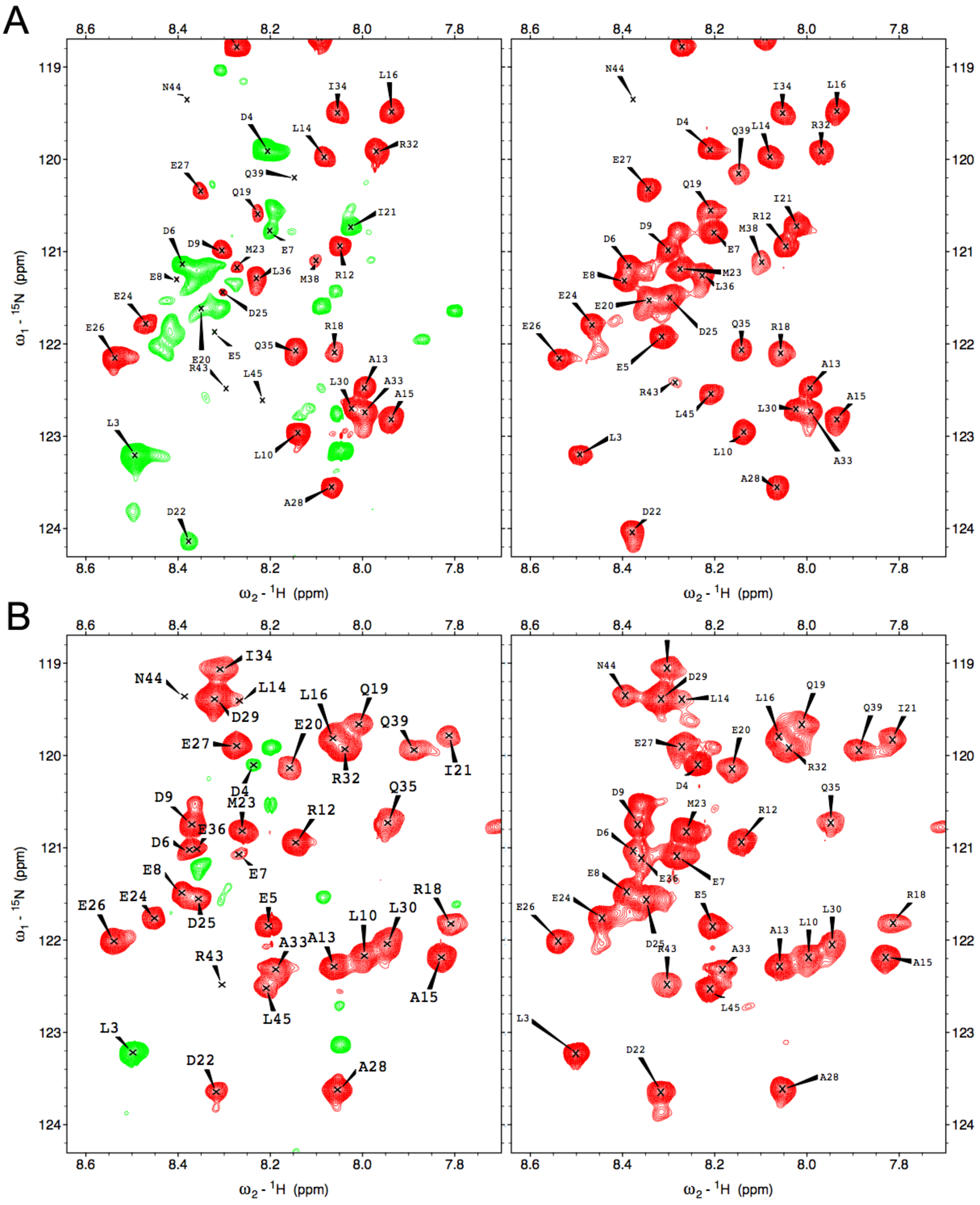

Figure S2. Steady-state hetero-nuclear ^{15}N-{^{1}H}-NOE experiments of AT3-UIM12 in the absence (A) and presence (B) of Ub. The spectra on the left were recorded with proton saturation, while those on the right were recorded without proton saturation. The ratios of the peak intensities represents the hetero-nuclear ^{15}N-{^{1}H}-NOE values for the backbone amides. Red, positive NOE; green, negative NOE.
