# Supplementary material for: Structural Transformation of the Tandem Ubiquitin-Interacting Motifs in Ataxin-3 and Their Cooperative Interactions with Ubiquitin Chains
Source: PLoS One. 2010 Oct 7;5(10):e13202. doi: 10.1371/journal.pone.0013202 (PMC2951365; doi:10.1371/journal.pone.0013202)
Supplement: Figure S3 — Pull-down Experiment Showing Bindings of AT3-UIM12 and AT3-UIM3 with Ub. The GST pull-down experiments were carried out between GST-Ub and AT3-UIMs, and detected by Coomassie staining and Western blotting with anti-His antibody. AT3-UIM12 pulled down by GST-Ub is indicated by an asterisk. AT3-UIM12 refers to the tandem UIMs of AT3 (residues 222–263); and AT3-UIM3 denotes the third UIM motif (residues 335–354). (0.08 MB PDF) [file pone.0013202.s005.pdf]

Figure S3

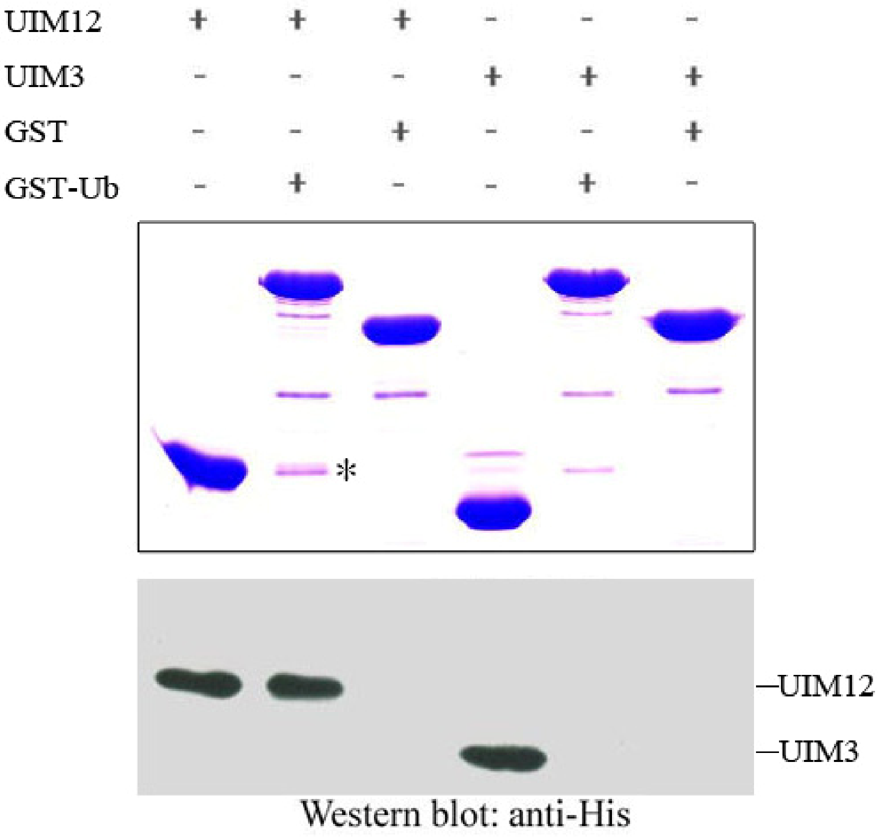

Figure S3. Pull-down Experiment Showing Bindings of AT3-UIM12 and AT3-UIM3 with Ub. The GST pull-down experiments were carried out between GST-Ub and AT3-UIMs, and detected by Coomassie staining and Western blotting with anti-His antibody. AT3-UIM12 pulled down by GST-Ub is indicated by an asterisk. AT3-UIM12 refers to the tandem UIMs of AT3 (residues 222 – 263); and AT3-UIM3 denotes the third UIM motif (residues 335 – 354).
