# Supplementary material for: Structural Transformation of the Tandem Ubiquitin-Interacting Motifs in Ataxin-3 and Their Cooperative Interactions with Ubiquitin Chains
Source: PLoS One. 2010 Oct 7;5(10):e13202. doi: 10.1371/journal.pone.0013202 (PMC2951365; doi:10.1371/journal.pone.0013202)
Supplement: Figure S4 — NMR Titration Showing the Interaction of Tandem AT3-UIM12 with Ub. (A) Traces for the chemical shift changes of the representative residues of AT3-UIM12 upon Ub titration. The peaks are colored from red (UIM/Ub = 1∶0) to coral (1∶6). (B) Titration curves for two equivalent residues, Ser236 in UIM1 and Ser256 in UIM2 (left panel), and Ala232 and Ala252 (right), in tandem AT3-UIM12 upon increasing amount of Ub. Note that the Δδ values of the residues in UIM2 moiety upon Ub titration reach the plateau with lower amount of Ub than the corresponding ones in UIM1. (0.14 MB PDF) [file pone.0013202.s006.pdf]

Figure S4

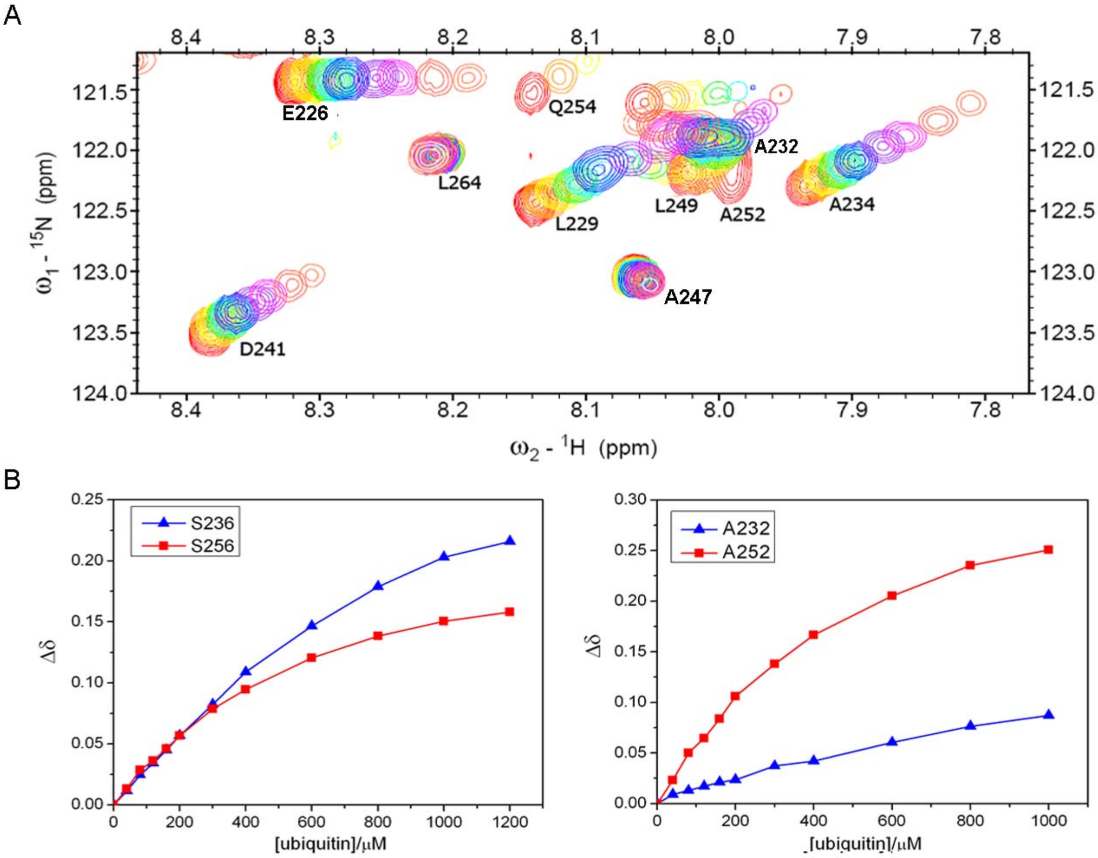

Figure S4. NMR Titration Showing the Interaction of Tandem AT3-UIM12 with Ub. (A) Traces for the chemical shift changes of the representative residues of AT3-UIM12 upon Ub titration. The peaks are colored from red (UIM/Ub = 1:0) to coral (1:6). (B) Titration curves for two equivalent residues, Ser236 in UIM1 and Ser256 in UIM2 (left panel), and Ala232 and Ala252 (right), in tandem AT3-UIM12 upon increasing amount of Ub. Note that the Δδ values of the residues in UIM2 moiety upon Ub titration reach the plateau with lower amount of Ub than the corresponding ones in UIM1.
