# Supplementary material for: Structural Transformation of the Tandem Ubiquitin-Interacting Motifs in Ataxin-3 and Their Cooperative Interactions with Ubiquitin Chains
Source: PLoS One. 2010 Oct 7;5(10):e13202. doi: 10.1371/journal.pone.0013202 (PMC2951365; doi:10.1371/journal.pone.0013202)
Supplement: Figure S5 — Interaction of the Linker Mutants of AT3-UIM12 with Ub. The titration curves for wild-type AT3-UIM12 and its linker mutants (I240A, I240P, Q238E/E239T) binding with Ub are plotted using the chemical shift changes of Leu71 of Ub as a representative. The dissociation constants for these mutants binding with Ub are listed in Table 1. (0.03 MB PDF) [file pone.0013202.s007.pdf]

Figure S5

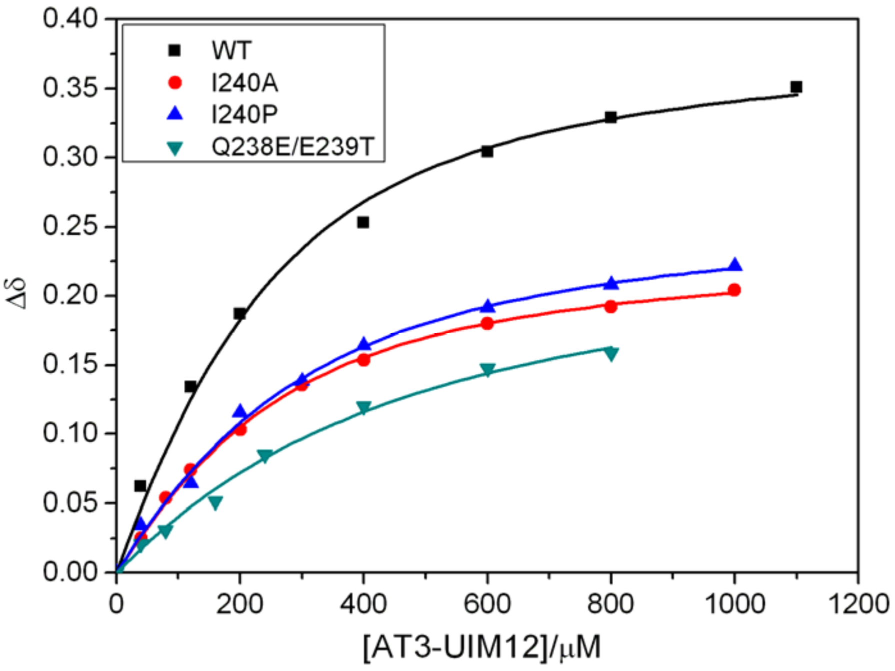

Figure S5. Interaction of the Linker Mutants of AT3-UIM12 with Ub. The titration curves for wild-type AT3-UIM12 and its linker mutants (I240A, I240P, Q238E/E239T) binding with Ub are plotted using the chemical shift changes of Leu71 of Ub as a representative. The dissociation constants for these mutants binding with Ub are listed in Table 1.
