# Supplementary material for: Structural Transformation of the Tandem Ubiquitin-Interacting Motifs in Ataxin-3 and Their Cooperative Interactions with Ubiquitin Chains
Source: PLoS One. 2010 Oct 7;5(10):e13202. doi: 10.1371/journal.pone.0013202 (PMC2951365; doi:10.1371/journal.pone.0013202)
Supplement: Figure S6 — Pull-down Experiments Showing the Interactions of Tandem UIM12 and Individual UIMs of AT3 with Different polyUb Chains. The Pull-down experiments were performed on various lengths of polyUb (Ub1∼7, K48- or K63-linked) with GST-fused UIM1, UIM2 or UIM12. The protein bands were detected by Western blotting with an anti-Ub antibody. (0.13 MB PDF) [file pone.0013202.s008.pdf]

Figure S6

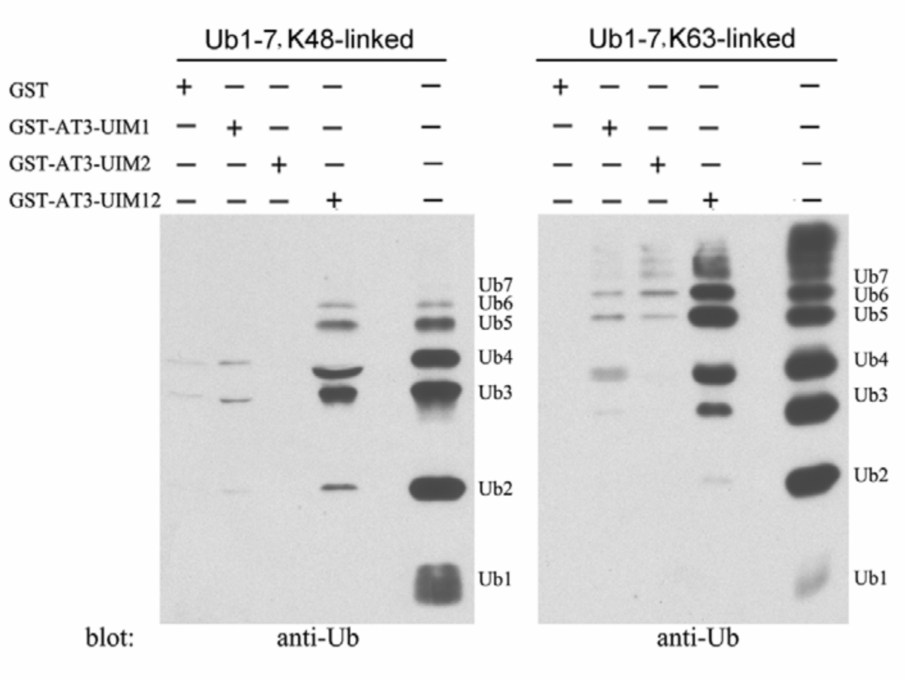

Figure S6. Pull-down Experiments Showing the Interactions of Tandem UIM12 and Individual UIMs of AT3 with Different polyUb Chains. The Pull-down experiments were performed on various lengths of polyUb (Ub1~7, K48- or K63-linked) with GST-fused UIM1, UIM2 or UIM12. The protein bands were detected by Western blotting with an anti-Ub antibody.
