# Supplementary material for: Structural Transformation of the Tandem Ubiquitin-Interacting Motifs in Ataxin-3 and Their Cooperative Interactions with Ubiquitin Chains
Source: PLoS One. 2010 Oct 7;5(10):e13202. doi: 10.1371/journal.pone.0013202 (PMC2951365; doi:10.1371/journal.pone.0013202)
Supplement: Figure S7 — Interactions of Tandem AT3-UIM12 with Different Forms of Ub by NMR Titrations. (A) Titration of 15N-labeled AT3-UIM12 with monoUb. (B) Titration of 15N-labeled AT3-UIM12 with linear diUb. (C) Titration of 15N-labeled AT3-UIM12 with K48-linked diUb. With the progress of K48-linked diUb titration, some peaks in the 1H-15N HSQC spectra experience large chemical shift changes and peak broadening. (D) & (E) Titration curves for two representative peaks (Q230 and S236) showing distinct binding affinities for monoUb, linear diUb and K48-linked diUb. (0.08 MB PDF) [file pone.0013202.s009.pdf]

Figure S7

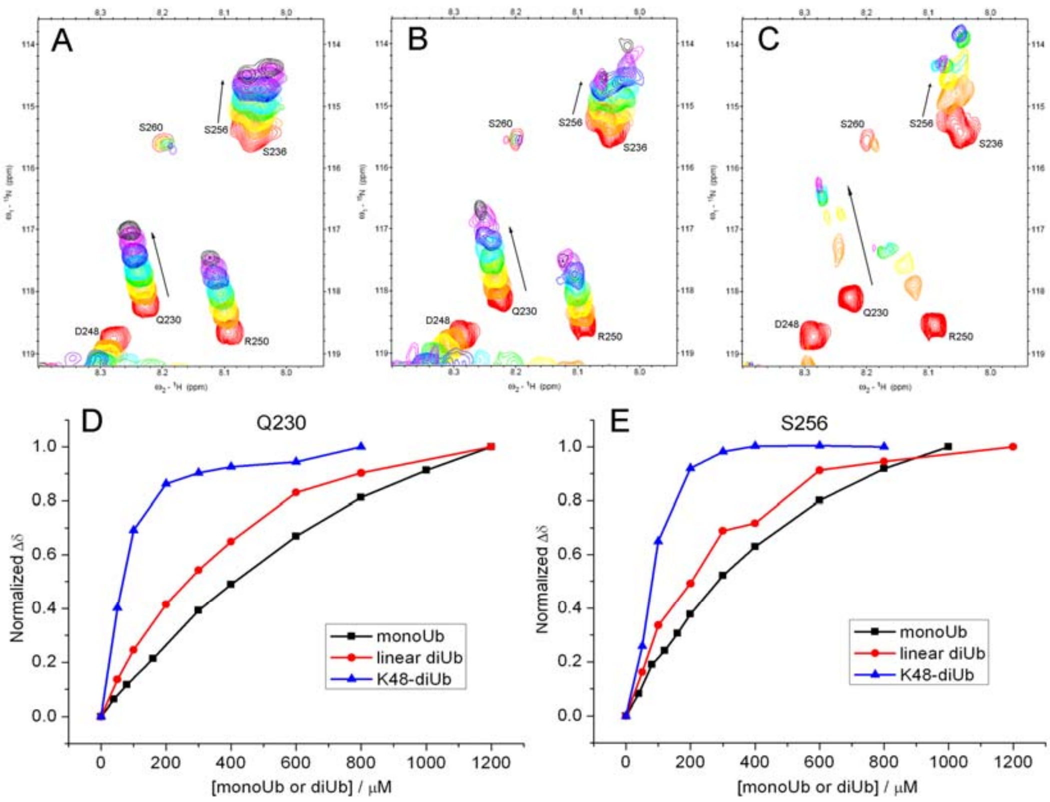

Figure S7. Interactions of Tandem AT3-UIM12 with Different Forms of Ub by NMR Titrations. (A) Titration of ^{15}N-labeled AT3-UIM12 with monoUb. (B) Titration of ^{15}N-labeled AT3-UIM12 with linear diUb. (C) Titration of ^{15}N-labeled AT3-UIM12 with K48-linked diUb. With the progress of K48-linked diUb titration, some peaks in the ^{1}H-^{15}N HSQC spectra experience large chemical shift changes and peak broadening. (D) & (E) Titration curves for two representative peaks (Q230 and S236) showing distinct binding affinities for monoUb, linear diUb and K48-linked diUb.
